# Systems immunology-based drug repurposing framework to target inflammation in atherosclerosis

**DOI:** 10.1038/s44161-023-00278-y

**Published:** 2023-06-08

**Authors:** Letizia Amadori, Claudia Calcagno, Dawn M. Fernandez, Simon Koplev, Nicolas Fernandez, Ravneet Kaur, Pauline Mury, Nayaab S Khan, Swathy Sajja, Roza Shamailova, Yannick Cyr, Minji Jeon, Christopher A. Hill, Peik Sean Chong, Sonum Naidu, Ken Sakurai, Adam Ali Ghotbi, Raphael Soler, Natalia Eberhardt, Adeeb Rahman, Peter Faries, Kathryn J. Moore, Zahi A. Fayad, Avi Ma’ayan, Chiara Giannarelli

**Affiliations:** 1 Department of Medicine, Division of Cardiology, NYU Cardiovascular Research Center, New York, NY USA; 2 The Icahn Institute for Genomics and Multiscale Biology, Icahn School of Medicine at Mount Sinai, New York, NY USA; 3 BioMedical Engineering and Imaging Institute, Icahn School of Medicine at Mount Sinai, New York, NY USA; 4 Department of Diagnostic, Molecular and Interventional Radiology, Icahn School of Medicine at Mount Sinai, New York, NY USA; 5 Department of Medicine, Division of Cardiology, Icahn School of Medicine at Mount Sinai, New York, NY USA; 6 Mount Sinai Center for Bioinformatics, Department of Pharmacological Sciences, Icahn School of Medicine at Mount Sinai, New York, NY USA; 7 Precision Immunology Institute, Icahn School of Medicine at Mount Sinai, New York, NY USA; 8 Department of Surgery, Vascular Division, Icahn School of Medicine at Mount Sinai, New York, NY USA; 9 Department of Pathology; NYU Grossman School of Medicine, NYU Langone Health, New York, NY USA

**Keywords:** Systems analysis, Cardiology, Immunotherapy, Drug discovery, Dyslipidaemias

## Abstract

The development of new immunotherapies to treat the inflammatory mechanisms that sustain atherosclerotic cardiovascular disease (ASCVD) is urgently needed. Herein, we present a path to drug repurposing to identify immunotherapies for ASCVD. The integration of time-of-flight mass cytometry and RNA sequencing identified unique inflammatory signatures in peripheral blood mononuclear cells stimulated with ASCVD plasma. By comparing these inflammatory signatures to large-scale gene expression data from the LINCS L1000 dataset, we identified drugs that could reverse this inflammatory response. Ex vivo screens, using human samples, showed that saracatinib—a phase 2a-ready SRC and ABL inhibitor—reversed the inflammatory responses induced by ASCVD plasma. In *Apoe*^−/−^ mice, saracatinib reduced atherosclerosis progression by reprogramming reparative macrophages. In a rabbit model of advanced atherosclerosis, saracatinib reduced plaque inflammation measured by [^18^F]fluorodeoxyglucose positron emission tomography–magnetic resonance imaging. Here we show a systems immunology-driven drug repurposing with a preclinical validation strategy to aid the development of cardiovascular immunotherapies.

## Abstract (web summary)

Amadori et al. show that plasma from patients with atherosclerosis triggers unique inflammatory transcriptomic and signaling signatures in peripheral blood mononuclear cells, which were used for selecting drugs that may be repurposed for the treatment of atherosclerosis.

## Main

ASCVD is the leading cause of death worldwide^1,2^, but the development of new cardiovascular drugs has lagged compared with the advancements made for other complex diseases conditions, such as cancer^3^. The current standard of care for ASCVD is to lower lipid levels and control other cardiovascular risk factors (such as diabetes, hypertension)^4^, but these approaches do not directly address the underlying inflammatory mechanisms of the disease^5^. Indeed, since the discovery of lipid-lowering statins^6^ and the recent PCSK9 inhibitors^7^, drug innovation in the field has been stagnant. This is in part due to the failures of traditional drug discovery efforts^8^ and the substantial investments required for large, outcome-driven phase 3 clinical trials with long-term follow-up for outcomes in individuals with ASCVD^9^. Immunomodulatory treatments are a promising approach to reduce the residual risk of stroke and myocardial infarction in individuals with ASCVD.

Drug repurposing is a cost-effective approach to rapidly transition existing drugs into the clinic for new indications^3^. Immunomodulatory drug repurposing studies have proven successful in recent years. For example, the knowledge that interleukin 1β (IL-1β) drives inflammation in ASCVD led to the successful design of the Canakinumab Anti-inflammatory Thrombosis Outcome Study^10^. In 2017, this seminal study proved that targeting inflammation reduces the risk for secondary cardiovascular events in patients, but US Food and Drug Administration approval for the use of IL-1β humanized neutralizing antibody canakinumab was not granted because the data were considered insufficient to justify routine use in patients with ASCVD. In 2019, the Colchicine Cardiovascular Outcomes Trial^11^ showed that the anti-inflammatory drug colchicine achieved similar efficacy on cardiovascular outcomes for patients with ASCVD.

However, other drug repurposing-based clinical trials were either unsuccessful or showed that a one-size-fits-all immunotherapeutic approach is unattainable due to variability in patient responses. For example, the Cardiovascular Inflammation Reduction Trial^12^ showed no efficacy of low-dose methotrexate, the gold-standard therapy for rheumatic arthritis, in patients with ASCVD. Moreover, patient outcomes on colchicine treatment proved more mixed than initially recognized, whereas low-dose colchicine reduced composite cardiovascular endpoints in patients with stable coronary artery disease (CAD) in two trials^13,14^. In the Colchicine in Patients with Acute Coronary Syndromes trial, patients had higher mortality and experienced no reduction in cardiovascular outcomes at 12 months^15^. These studies suggest that further investigation of immunomodulatory therapies in ASCVD is warranted.

Multifactorial disorders like ASCVD are modulated by complex gene and protein regulatory networks that span the interactions between different cell types^16,17^. The advent of single-cell analyses and systems biology have revealed heterogeneous immune alterations in the blood and in atherosclerotic vascular tissues of patients, and uncovered immune cell transcriptional alterations in plaques^18,19^. Harnessing system-level analyses offers the promise of discovering drugs that may restore dysregulated immune responses in ASCVD. Here we present a path to a drug repurposing approach that combines innovative systems immunology-driven drug repurposing with a functional screen that is applied directly to human samples. In conjunction with a rigorous preclinical validation platform in animal models, this system can aid the clinical translation of existing drugs with new cardiovascular indications tailored to individual patients.

## Results

### Phospho-CyTOF identifies immune alterations in patients with atherosclerosis

**Fig. 1:**
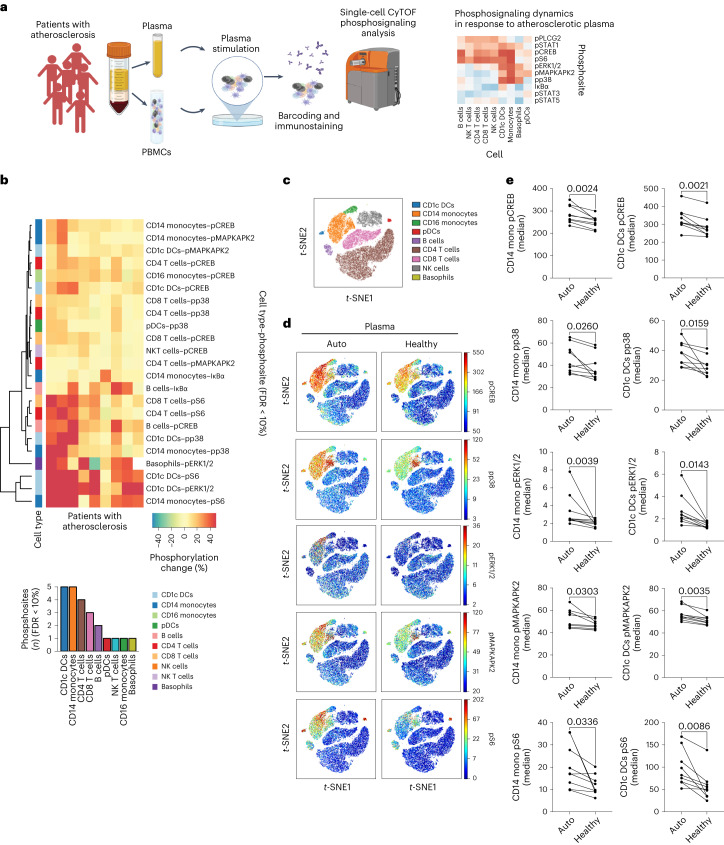
Single-cell mass cytometry reveals signaling dynamics of human PBMCs from patients with atherosclerosis exposed to autologous plasma. **a**, Experimental design. PBMCs and plasma were isolated from peripheral venous blood of patients with ASCVD (*n* = 9 biologically independent samples, 5 men). Healthy plasma was isolated from healthy donors (*n* = 9 biologically independent samples). Ex vivo stimulation of patients’ PBMCs with their autologous plasma was compared with stimulation using pooled healthy plasma, and intracellular signaling activation was analyzed by mass cytometry (CyTOF). This figure was created with Biorender.com. **b**, Biclustered heat map of filtered cell type–phosphoprotein data (FDR < 10%) shows significant activation of intracellular signaling (percentage phosphorylation change, auto versus healthy) in response to autologous (auto) versus pooled healthy plasma (healthy) measured as intracellular protein phosphorylation (*n* = 9). CD14^+^ monocyte and CD1c^+^ DCs were the most responsive cells, as shown by the number of phosphosites activated by atherosclerotic plasma. **c**, viSNE plot of PBMCs from patients with atherosclerosis shows major immune cell subsets based on canonical expression markers. **d**, Single-cell signaling patterns in response to autologous atherosclerotic (*n* = 9) or pooled healthy (*n* = 9) plasma were visualized across this immune map. **e**, Dot plots show the effect of autologous plasma (*n* = 9) versus pooled healthy plasma (*n* = 9) on the phosphorylation of intracellular kinases in CD14^+^ monocytes and CD1c^+^ DCs. *P* values were determined by two-tailed paired *t*-test. mono, monocyte; NK, natural killer; *t-*SNE, *t*-distributed stochastic neighbor embedding.

**Extended Data Fig. 1:**
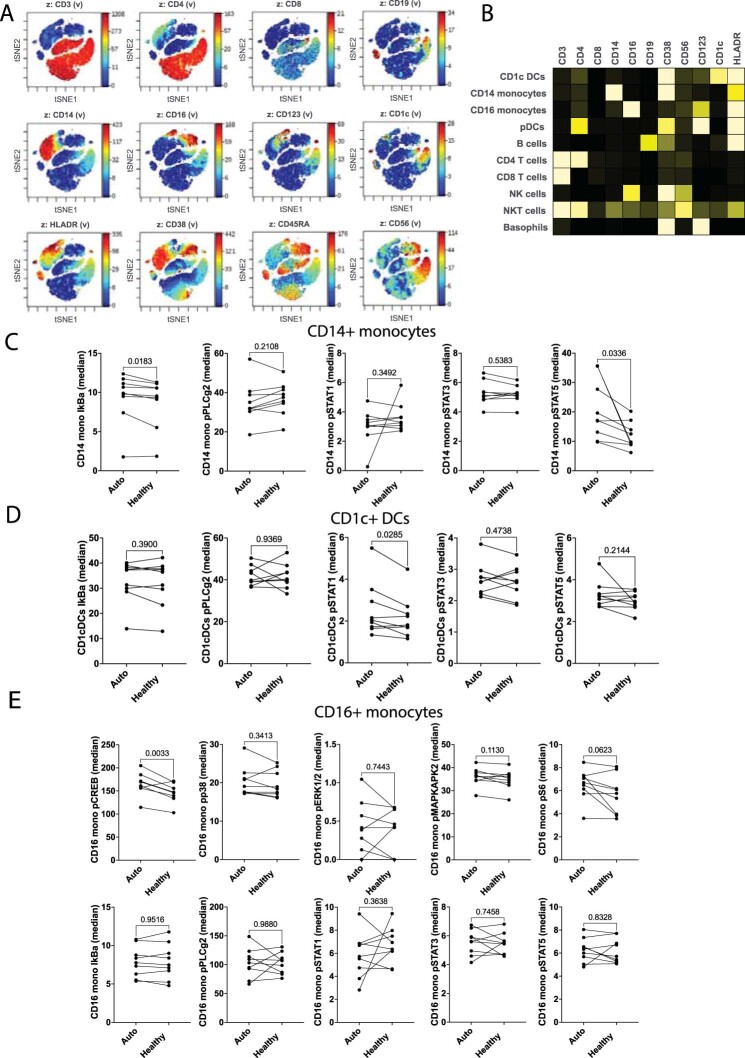
Ex-vivo plasma-stimulated PBMCs from atherosclerotic patients were stained with a panel of antibodies and analyzed by phospho-CyTOF. **A**. Expression of surface canonical markers using viSNE in Cytobank. The distribution of each of the clustering parameters is presented as a color scale in z-dimension for manual identification of each cluster immune population in Fig. 1. **B**. Expression of surface canonical markers visualized using a heat map. **C**. Dot plots show the effect of autologous plasma vs. healthy plasma on the phosphorylation of intracellular kinases in CD14^+^ monocytes (n = 9 biologically independent samples; males=5). P values were determined by paired two-tailed t-test. **D**. Dot plots show the effect of autologous plasma vs. healthy plasma on the phosphorylation of intracellular kinases in CD1c^+^ DCs (n = 9 biologically independent samples; males=5). P values were determined by paired two-tailed t-test. **E**. Dot plots show the effect of autologous plasma vs. healthy plasma on the phosphorylation of intracellular kinases in CD16^+^ monocytes (n = 9 biologically independent samples; males=5). P values were determined by paired two-tailed t-test.

To characterize functional dysregulation of immune cells in human atherosclerosis, we isolated peripheral blood mononuclear cells (PBMCs) from patients with carotid atherosclerosis (Supplementary Table 1) and exposed them to either autologous plasma (referred to as atheroplasma or atherosclerotic plasma) or plasma from healthy donors (referred to as healthy plasma). Using phospho-cytometry by time-of-flight (phospho-CyTOF), a mass cytometry method to study intracellular phospho signaling pathways at the single-cell level, we then interrogated the activation of major immune cell signaling pathways across all main immune populations (Fig. 1a). Using viSNE, a visualization tool for high-dimensional single cell data, we visualized ten major immune cell populations (B cells, basophils, CD1c^+^ dendritic cells (DCs), CD4^+^ T cells, CD8^+^ T cells, CD14^+^ and CD16^+^ monocytes, natural killer cells, natural killer T cells and plasmacytoid DCs) on the basis of canonical marker expression patterns (Extended Data Fig. 1a,b). Next, to identify intracellular signaling pathways activated within each population, we quantified the phosphorylation of ten intracellular proteins (IκBɑ (nuclear factor of κ light chain polypeptide gene enhancer in B cells inhibitor-ɑ), CREB (cAMP-response element binding protein), ERK1/2 (extracellular signal-regulated kinase 1 and 2), MAPKAPK2 (mitogen-activated protein (MAP) kinase-activated protein kinase 2), p38 (p38 MAP kinase), PLCG2 (phospholipase Cγ2), S6 (ribosomal protein S6), STAT1 (signal transducer and activator of transcription 1), STAT3 and STAT5) across this immunological map. Data were integrated to derive 100 cell type–phosphoprotein pairs that were compared across each condition, revealing the greatest immune activation in CD14^+^ monocytes and CD1c^+^ DCs (Fig. 1b). Specifically, compared with healthy plasma, exposure to autologous atherosclerotic plasma induced the phosphorylation of CREB, p38, ERK1/2, MAPKAPK2 and S6 in CD14^+^ monocytes and CD1c^+^ DCs (Fig. 1c–e, Extended Data Fig. 1c–e). Other immune cell types responded to autologous atherosclerotic plasma, including CD4^+^ and CD8^+^ T cells, but both the number of activated phosphosites and the magnitude of their activation was lower than in CD14^+^ monocytes and CD1c^+^ DCs (Fig. 1b). These results suggest that, in patients with atherosclerotic disease, plasma from these patients induces a strong and specific innate immune cell signaling responses in circulating inflammatory cells.

### Atherosclerotic plasma shapes inflammatory responses

**Fig. 2:**
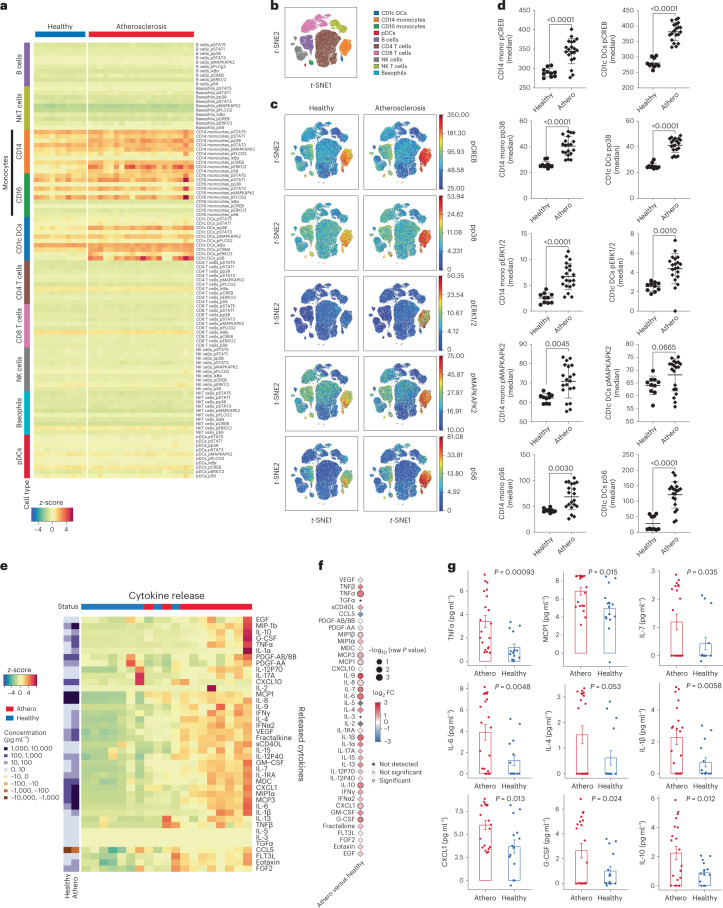
Multiplexed mass cytometry of intracellular signaling and cytokine expression profile mark the response of healthy immune cells to plasma from patients with atherosclerosis. **a**, Heat map of mass cytometry data, ordered by stimulatory plasma condition and immune cell types, highlights the activation of specific intracellular markers in monocytes and CD1c^+^ DCs in response to plasma from patients with atherosclerosis (athero; *n* = 20 biologically independent samples, 10 men) or healthy plasma (*n* = 10 biologically independent samples). **b**, viSNE plot of all major healthy PBMC cell types defined based on canonical expression patterns. **c**, Intracellular signaling patterns were visualized across this immune map in response to plasma from patients with atherosclerosis (*n* = 20) or healthy plasma (*n* = 10). **d**, Dot plots show the effect of plasma from patients with atherosclerosis (*n* = 20) versus healthy plasma (*n* = 10) on the phosphorylation of intracellular kinases in CD14^+^ monocytes and CD1c^+^ DCs. *P* values were determined by two-tailed unpaired *t*-test. Data are presented as mean ± s.d. **e**, Heat map of cytokines released by healthy PBMCs stimulated with atherosclerotic (red; *n* = 10 biologically independent samples, 5 men) versus healthy donor (blue; *n* = 9 biologically independent samples) plasma, with clustering based on standardized *z*-scores of cytokine values and corresponding concentrations (picograms per milliliter). **f**, Point plot of cytokines released by PBMCs stimulated with plasma from patients with atherosclerosis (*n* = 20 biologically independent samples, 10 men) versus healthy plasma (*n* = 15 biologically independent samples). *P* values were determined by unpaired two-tailed *t*-test. **g**, Bar graph with overlapping dots of significant cytokines released by healthy PBMCs stimulated with either atherosclerotic patient (red; *n* = 20) or healthy plasma (blue; *n* = 15). *P* values were determined by unpaired two-tailed *t*-test. Data are presented as mean ± s.e.m.

**Extended Data Fig. 2:**
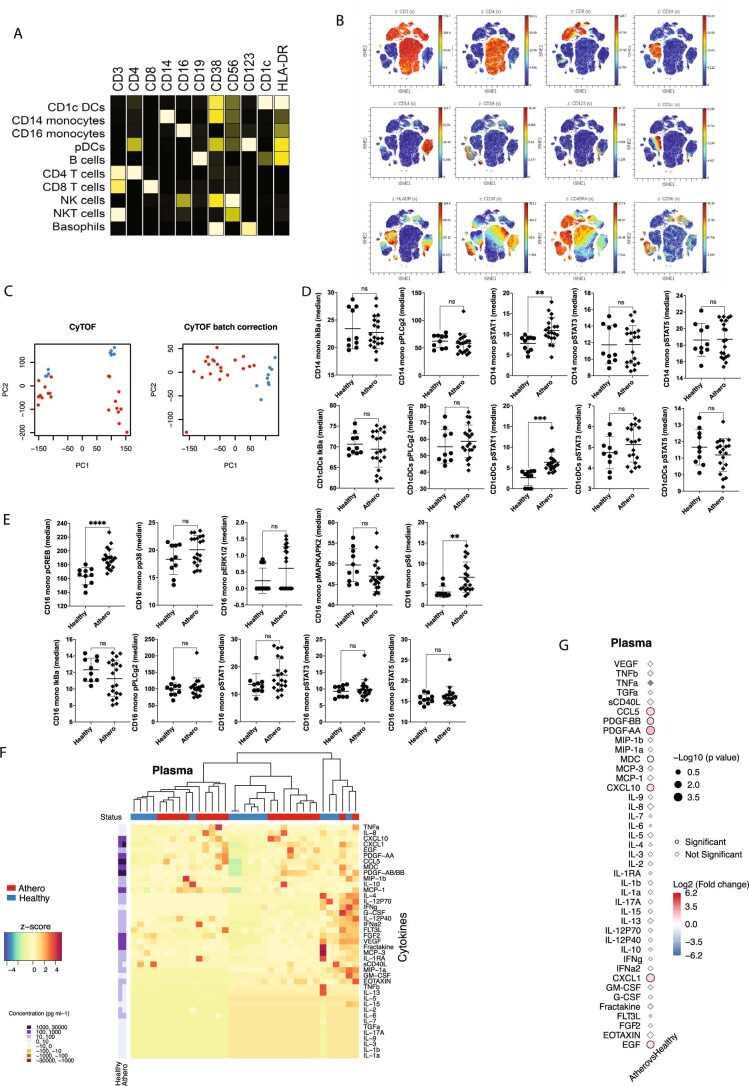
Ex-vivo stimulated healthy PBMCs were stained with a panel of antibodies and analyzed by CyTOF and Luminex. **A**. Expression of surface canonical markers using viSNE in Cytobank. The distribution of each of the clustering parameters is presented as a color scale in z-dimension for the manual identification of each cluster immune population in Fig. 2. **B**. Expression of surface canonical markers visualized using a heatmap. **C**. Batch correction of CyTOF data from 2 independent experiments used for the results of Fig. 2. Blue dots correspond to responses to healthy plasma (n = 10 biologically independent samples), red dots to atherosclerosis plasma (n = 20 biologically independent samples; males=10). **D**. Dot plots show the effect of atherosclerotic plasma (n = 20 biologically independent samples; males=10) vs. healthy plasma (n = 10 biologically independent samples) on the phosphorylation of intracellular kinases in CD14^+^ monocytes and CD1c^+^ DCs. P values were determined by unpaired two-tailed t-test. Data are presented as mean values +/- SD. **E**. Dot plots show the effect of atherosclerotic plasma (n = 20 biologically independent samples; males=10) vs. healthy plasma (n = 10 biologically independent samples) on the phosphorylation of intracellular kinases in CD16^+^ monocytes. P values were determined by unpaired t-test, two-tailed. Data are presented as mean values +/- SD. **F**. Heatmap of cytokine levels in plasma of atherosclerotic (red, n = 20 biologically independent samples; males=10) vs. healthy donors’ (blue, n = 15 biologically independent samples) plasma with clustering based on standardized z-scores of cytokine values and correspondent concentrations (pg/ml). **G**. Point plot of cytokines levels in plasma of PBMCs stimulated with atherosclerotic patient plasma (n = 20 biologically independent samples; males=10) vs healthy plasma (n = 15 biologically independent samples). P values were determined by unpaired two-tailed t-test.

To determine whether the functional responses seen in PBMCs from patients with atherosclerotic artery disease were dependent on plasma stimulation, we first investigated the immune response of healthy PBMCs to patients’ plasma. We exposed PBMCs isolated from healthy donors to either plasma of patients with atherosclerosis or healthy autologous plasma, and analyzed the activation of the same signaling pathways across all major immune subsets (Extended Data Fig. 2a–b). Notably, exposure of healthy PBMCs to patient plasma recapitulated the phosphorylation signature seen in stimulated patient PBMCs (Fig. 1) by primarily activating intracellular signaling in CD14^+^ monocytes and CD1c^+^ DCs (Fig. 2a and Extended Data Fig. 2c–e). Specifically, CD14^+^ monocytes and CD1c^+^ DCs exhibited the greatest immune activation, with increased phosphorylation of CREB, p38, ERK1/2, MAPKAPK2 and S6 in response to atherosclerotic plasma (Fig. 2b–d).

To further investigate this inflammatory response, we measured the baseline cytokine levels in healthy and patient plasma (Extended Data Fig. 2f,g), and then calculated the net effective release (Methods) of cytokines from PBMCs after ex vivo plasma stimulation (Fig. 2e–g). Exposure to atherosclerotic plasma induced a significant upregulation of several proinflammatory and proatherogenic cytokines (Fig. 2e–g), including IL-1β, whose inhibition reduces secondary cardiovascular events in patients who are at high risk postmyocardial infarction^10^, and IL-6, a biomarker of cardiovascular risk and a candidate therapeutic target in ongoing clinical trials^20^. Other released proatherogenic cytokines and factors included tumor necrosis factor-ɑ (TNFɑ), monocyte chemoattractant protein 1 (MCP1, also known as chemokine (C–C motif) ligand 2 (CCL2)), granulocyte colony stimulating factor (G-CSF), chemokine (C–X–C motif) ligand 1 (CXCL1; also known as growth-regulated oncogene (GRO)) and IL-7, a chemokine involved in monocyte recruitment to tissues^21^. The simultaneous release of antiatherogenic IL-10 (ref. ^22^) probably reflected immunoregulatory feedback.

**Fig. 3:**
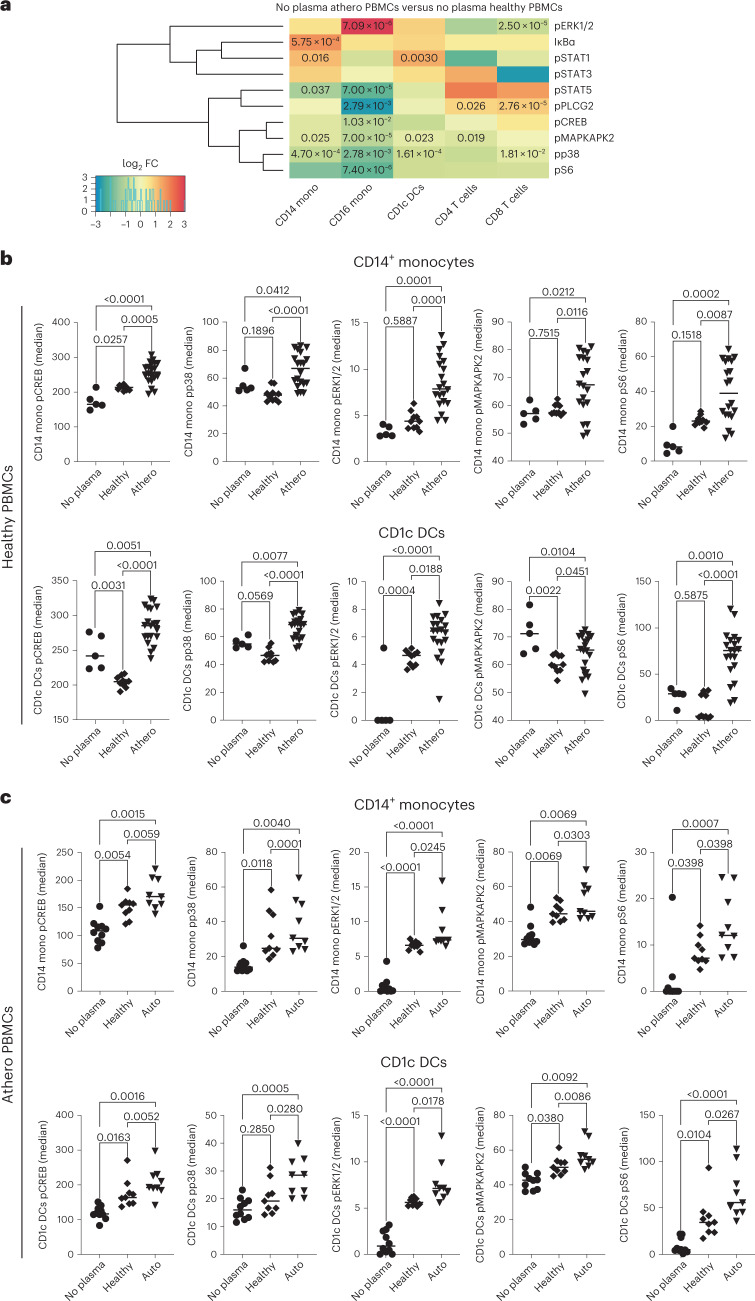
Single-cell mass cytometry reveals distinct resting and stimulated immune responses in PBMCs from patients with atherosclerosis and healthy donors. **a**, Heat map of mass cytometry data, ordered by immune cell types, highlights the activation of specific intracellular markers in unstimulated PBMCs from patients with atherosclerosis (no plasma athero PBMCs; *n* = 10 biologically independent samples, 5 men) versus unstimulated PBMCs from healthy donors (no plasma healthy PBMCs; *n* = 5 biologically independent samples). Clustering was based on standardized *z*-scores of median phosphoprotein values with absolute log_2_ FC > 0 considered upregulated with respect to healthy donors. Unpaired two-tailed *t*-test was used for significance. The Benjamini–Hochberg method was used for multiple correction (FDR < 0.05) and adjusted *P* values <0.05 were considered significant. **b**, Dot plots show the effect in PBMCs from healthy donors of atherosclerotic plasma (*n* = 20 biologically independent samples, 10 men) versus healthy plasma (*n* = 10 biologically independent samples) or no stimulation (no plasma; *n* = 5 biologically independent samples) on the phosphorylation of intracellular kinases in CD14^+^ monocytes and CD1c^+^ DCs. *P* values were determined by one-way ANOVA with Tukey’s post hoc test across all groups. **c**, Dot plots show the effect in PBMCs from autogolous plasma from patients with atherosclerosis (*n* = 9 biologically independent samples, 5 males) versus healthy plasma (*n* = 9 biologically independent samples) or no stimulation (*n* = 10 biologically independent samples, 5 men) on the phosphorylation of intracellular kinases in CD14^+^ monocytes and CD1c^+^ DCs. *P* values were determined by one-way ANOVA with Tukey’s post hoc test across all groups.

Having established the role of atherosclerotic plasma in driving the phosphorylation signature in stimulated PBMCs from both patients and healthy donors, we next investigated whether PBMCs from patients contained a pre-existing inflammatory signature as a result of their previous exposure to atherosclerotic plasma that would influence their immune response. Counterintuitively, baseline phosphorylation of most intracellular proteins (IκBɑ, CREB, ERK1/2, MAPKAPK2, p38, PLCG2, S6, STAT1, STAT3 and STAT5) was lower in unstimulated PBMCs from patients versus healthy donors, with the exception of phosphorylated IκBɑ (pIκBɑ) and pSTAT1 in CD14^+^ monocytes, pERK1/2 in CD16^+^ monocytes, pSTAT1 in CD1c^+^ DCs and pPLCG2 in T cells, which were all significantly activated in PBMCs from patients (Fig. 3a).

**Extended Data Fig. 3:**
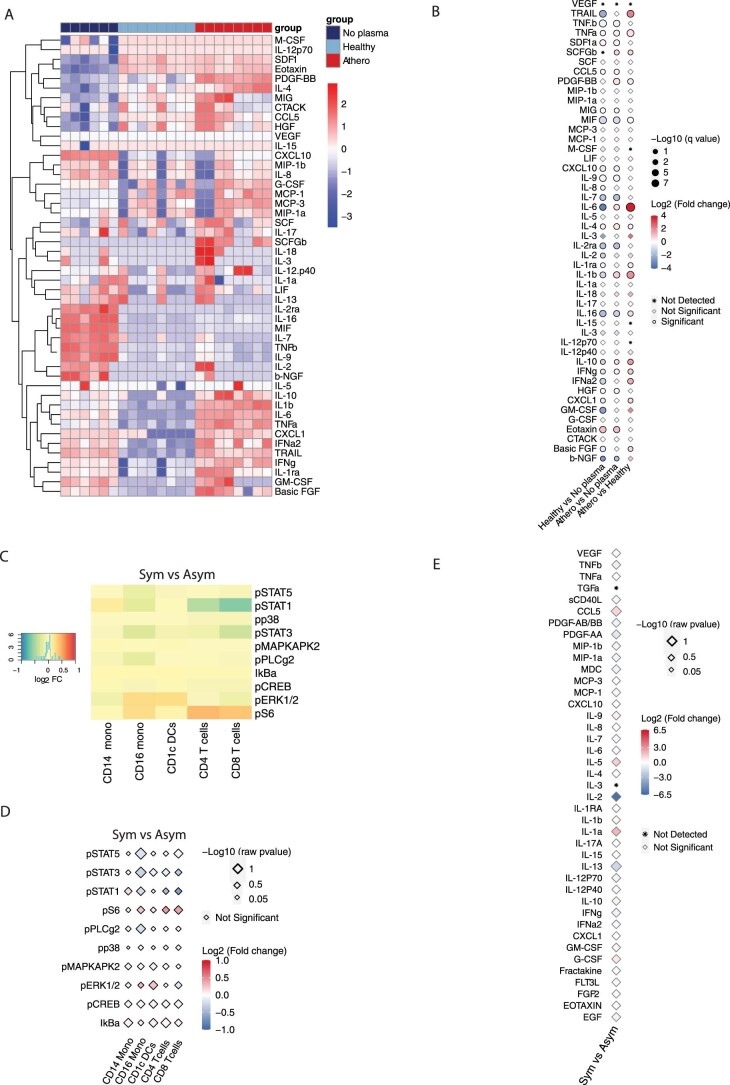
Immune responses to atheroplasma and healthy plasma versus baseline and to plasma from symptomatic or asymptomatic patients. **A**. Heatmap of cytokines released by healthy PBMCs stimulated with atherosclerotic (red, n = 8 samples/condition) vs. healthy donor (light blue, n = 8 samples/condition) plasma or not stimulated (no plasma, dark blue, n = 6 samples/condition), with clustering based on standardized z-scores of cytokine values. **B**. Point plot of cytokines released by PBMCs stimulated with atherosclerotic patient plasma (n = 8) vs healthy plasma (n = 8) or not stimulated cells (no plasma, n = 6). P-values were determined using unpaired t-test and corrected for multiple comparison using Benjamini-Hochberg (BH) method (FDR < 0.1). **C**. Heatmap of log_2_ fold change of phosphoproteins mass cytometry data, ordered by immune cell types, shows no significant difference in PBMCs stimulated with atherosclerotic plasma from symptomatic patients (sym, n = 10, males=5) vs asymptomatic patients (asym, n = 10, males=5). **D**. Point plot of phosphoproteins shows no significant difference in their expression in immune cell types in PBMCs stimulated with atherosclerotic plasma from symptomatic patients (sym, n = 10, males=5) vs asymptomatic patients (asym, n = 10, males=5). P-values were determined using unpaired t-test and corrected for multiple comparison using Benjamini-Hochberg (BH) method (FDR < 0.05). P < 0.05 was considered significant. **E**. Point plot shows no significant difference in cytokine released by PBMCs stimulated with atherosclerotic plasma from symptomatic patients (sym, n = 10, males=5) vs asymptomatic patients (asym, n = 10, males=5). P-values were determined using unpaired two-tailed t-test. P < 0.05 was considered significant.

Despite a lower baseline phosphosignature, CD14^+^ monocytes and CD1c^+^ DCs from both patients and healthy donors presented very similar phospho-CyTOF responses to atherosclerotic plasma (Fig. 3b,c), including the release of several proatherogenic cytokines such as IL-6 and IL-1β (Extended Data Fig. 3a,b). In contrast, exposure to healthy plasma highlighted divergent phosphosignatures (Fig. 3b,c and Supplementary Figs. 1 and 2). Specifically, stimulation of healthy PBMCs with autologous healthy plasma did not increase the phosphorylation of ERK1/2, MAPKAPK2, p38, or S6 in CD14^+^ monocytes, but did reduce pCREB and pMAPKAPK2 and increase pERK1/2 in CD1c^+^ DCs (Fig. 3b). Other immune cell populations showed either reduction or no effect on the phosphorylation of most kinases and transcription factors (TFs) (Supplementary Fig. 1). Moreover, autologous healthy plasma reduced the baseline secretion of several proatherogenic cytokines (that is, IL-6, IL1-β, interferon-γ (IFNγ), IFNɑ and macrophage migration inhibitory factor (MIF)) from healthy PBMCs (Extended Data Fig. 3a,b). Immune cells from patients responded differently and were instead activated by both healthy plasma and autologous atherosclerotic plasma; yet the magnitude of the immune response was greater in response to autologous atheroplasma (Fig. 3c and Supplementary Fig. 2).

To further elucidate the observed immune response to atheroplasma, we compared intracellular phosphorylation and cytokine release in response to plasma from symptomatic patients with a recent (<6 months) transient ischemic attack or stroke versus patients with no recent history of events (Supplementary Table 1). This analysis showed no difference in either phosphosignaling or cytokine release (Extended Data Fig. 3c–e).

Taken together, these results highlight that, despite key differences in baseline inflammatory signaling and response to healthy plasma between circulating immune cells of patients and healthy donors, plasma from patients triggers similar inflammatory responses in healthy and patient PBMCs. The stronger response seen in immune cells from patients suggests that inflammatory responses of circulating immune cells in patients with atherosclerotic disease are remarkably shaped by their interaction with plasma.

### Transcriptional signature induced by atherosclerotic plasma

**Fig. 4:**
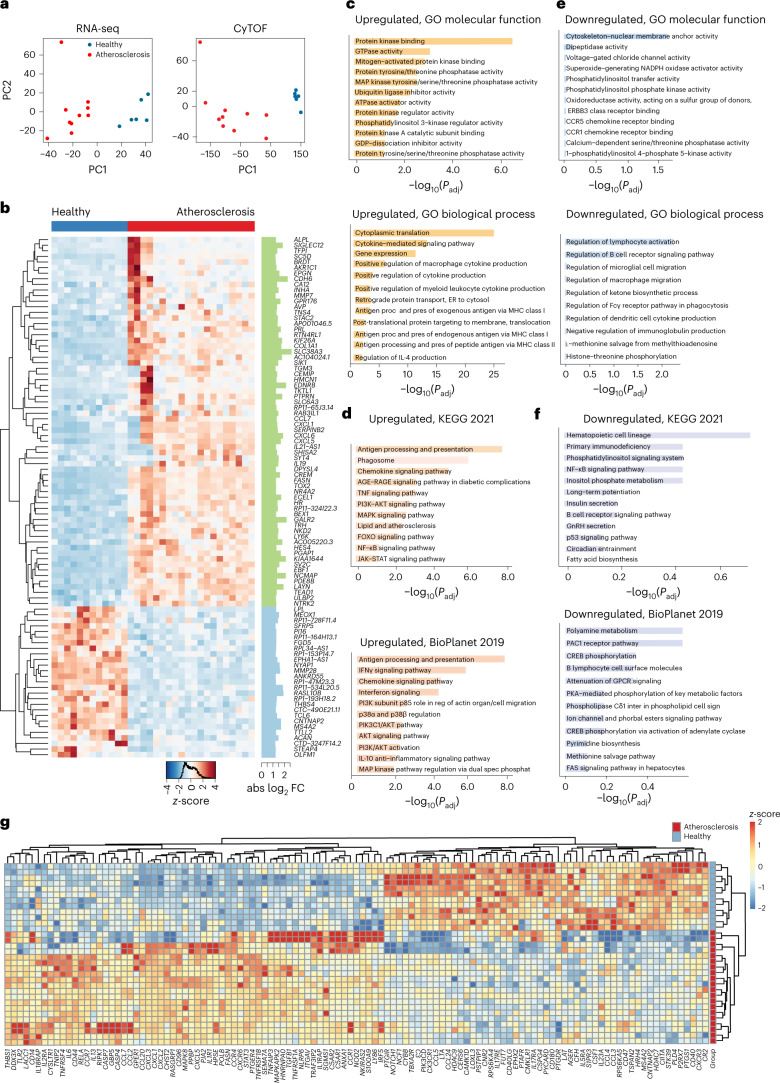
RNA-seq analysis of healthy PBMCs after plasma stimulation. **a**, PCA of RNA-seq and corresponding CyTOF data aggregated by cell type. **b**, Heat map of DEGs in response to atherosclerotic plasma (atherosclerosis; *n* = 20 biologically independent samples, 10 men) versus healthy plasma (*n* = 12 biologically independent samples) showing the *z*-score of transcripts with absolute log_2_ FC > 1.2 and normalized sequence counts >4. DESeq, Benjamini–Hochberg, *P* < 0.05. **c**, Enriched GO molecular function and GO biological processes of the significant upregulated genes in response to atherosclerotic plasma versus healthy plasma. **d**, Enriched KEGG and BioPlanet signaling pathways of the significant upregulated genes in response to atherosclerotic plasma versus healthy plasma. **e**, Enriched GO molecular function and GO biological processes of the significant downregulated genes in response to atherosclerotic plasma versus pooled healthy plasma. **f**, Enriched KEGG and BioPlanet signaling pathways of the significant downregulated genes in response to atherosclerotic plasma versus pooled healthy plasma. Adjusted *P* values in **c**–**f** were obtained using Fisher’s exact test and the Benjamini–Hochberg method. **g**, Heat map of a subnetwork of 227 DEGs associated with inflammatory response (GO:0006954) in response to atherosclerotic plasma versus pooled healthy plasma showing the *z*-score of transcripts with absolute log_2_ FC > 1.2 and normalized sequence counts >4. abs, absolute; AGE, advanced glycation end product; ER, endoplasmic reticulum; GnRH, gonadotropin-releasing hormone; inter, interaction; *P*_adj_, adjusted *P* value; PC, principal component; pres, presenting; proc, processing; RAGE, receptor for advanced glycation end product; reg, regulation; spec phosphat, specific phosphatase.

To investigate the transcriptional changes associated with the phosphosignaling induced by atherosclerotic plasma, we performed RNA-sequencing (RNA-seq) profiling of the same healthy PBMCs stimulated with the same atherosclerotic or healthy donor plasma. Principal component analysis (PCA) of all mapped genes showed a clear separation between PBMCs stimulated with plasma from patients and healthy donors, consistent with the phospho-CyTOF results (Fig. 4a). Differential gene expression analysis identified 4,823 differentially expressed genes (DEGs; false discovery rate (FDR) = 0.05) with 2,377 upregulated and 2,446 downregulated genes indicating a substantial transcriptional reprogramming induced by atherosclerotic plasma (Fig. 4b). Gene set enrichment analysis of the upregulated genes using Enrichr^23^ identified Gene Ontology (GO) molecular function terms consistent with the increased phosphorylation of PI3K (phosphatidylinositol 3-kinase) and MAP kinases identified by the phospho-CyTOF profiling (Fig. 4c). Other molecular functions identified for the upregulated gene set were GTPase activity and GDP-dissociation inhibitory activity, which are involved in the expression of inflammatory cytokines, including MCP1 and IL-6. Other enriched GO biological processes included the cytokine signaling pathway, regulation of cytokine production in macrophages and regulation of IL-4 production. Retrograde protein transport, endoplasmic reticulum-to-cytosol transport and processes involved in the secretion of cytokines from immune cells were also upregulated. Kyoto Encyclopedia of Genes and Genomes (KEGG) and BioPlanet signaling pathway analyses of the upregulated genes identified a collection of pathways with well-established proatherogenic functions, including phagosome, lipid and atherosclerosis, chemokine signaling pathways, TNF signaling, nuclear factor-κB (NF-κB) signaling pathway, IFN signaling and the activation of PI3K–AKT, MAPK and p38 signaling pathways (Fig. 4d). Enrichment analysis of the downregulated genes identified enrichment for gene sets, such as those involved in cytoskeleton–nuclear membrane anchor activity, which are essential for cell migration, phagocytosis, activation and regulation of lymphocyte activation and signaling pathways, including CREB phosphorylation—a key TF that was phosphorylated following plasma stimulation as measured by CyTOF (Fig. 4e,f).

**Extended Data Fig. 4:**
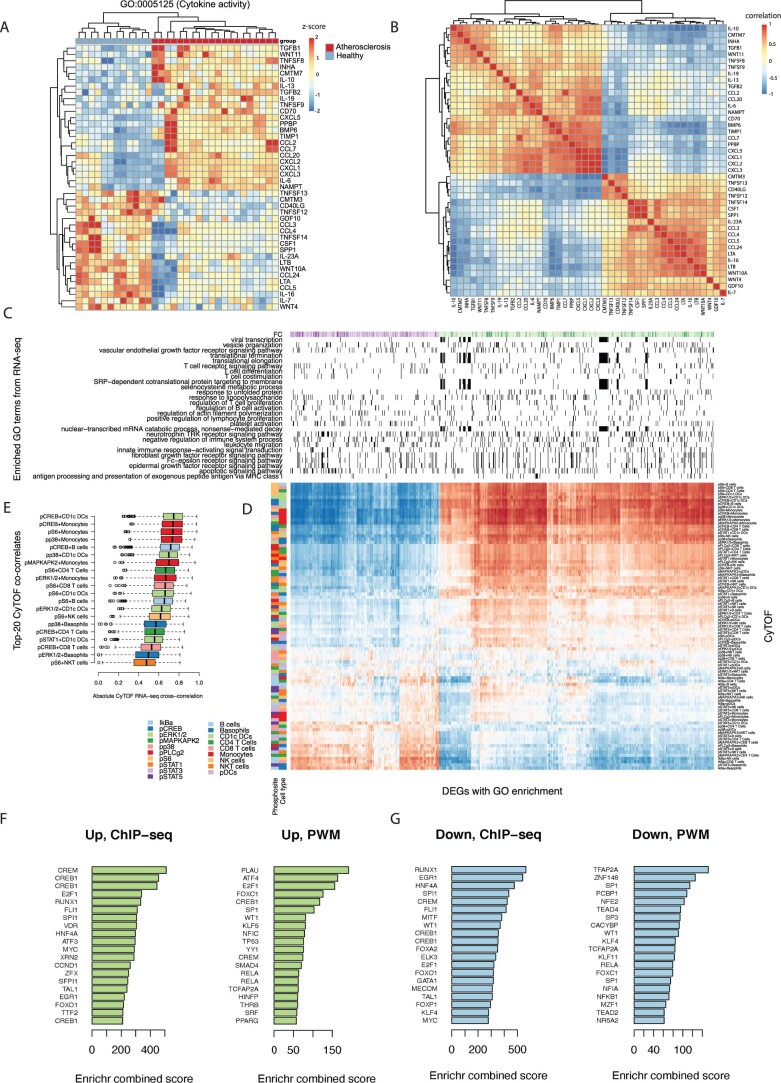
Integrative cross-correlation analysis of RNA-seq and aggregated mass cytometry data corroborated the atherosclerotic plasma-driven inflammatory signature and intracellular signaling. **A**. Heatmap of a sub-network of cytokine DEGs in response to atherosclerotic plasma (atherosclerosis, n = 20 biologically independent samples, males=10) vs. pooled healthy plasma (healthy, n = 12 biologically independent samples), showing z-score of transcripts with absolute log_2_ fold change >1.2 and normalized sequence counts >4 associated with cytokine activity (GO:0005125). **B**. Co-expression Pearson correlation analysis of DEGs in response to atherosclerotic plasma (atherosclerosis, n = 20 biologically independent samples, males=10) vs. pooled healthy plasma (healthy, n = 12 biologically independent samples), filtered for cytokine activity (GO:0005125). **C**. Enriched GO terms from gene expression data in response to atherosclerotic plasma (n = 20 biologically independent samples; males=10) vs. pooled healthy plasma (n = 12 biologically independent samples). **D**. Hierarchically ordered heatmap of Pearson’s correlations between gene expression and phosphoprotein-cell type pairs in response to atherosclerotic plasma (n = 20 biologically independent samples, males=10) vs. pooled healthy plasma (healthy, n = 12 biologically independent samples). Only DEGs in healthy PBMCs, in response to atherosclerotic plasma, belonging to the enriched GO terms are included. **E**. Pairs of phosphoprotein and cell-type with the highest median cross-correlation with RNA-seq data. Box plots showing the median and range (min to max). **F**. Enriched TFs obtained by analyzing upregulated DEGs in healthy PBMCs, in response to atherosclerotic plasma, against ChIP-seq libraries and position weight matrix (PWM) predictions. **G**. Enriched TFs obtained by analyzing downregulated DEGs in healthy PBMCs, in response to atherosclerotic plasma, against ChIP-seq libraries and position weight matrix (PWM) predictions.

The analysis of 227 DEGs associated with the inflammatory response (GO:0006954) identified 116 genes upregulated in response to atherosclerotic plasma, including *CCL2* (encoding MCP1) and *IL6* (Fig. 4g). Other proinflammatory and proatherogenic genes upregulated by atherosclerotic plasma were *S100A9* (encoding S100 calcium-binding protein A9) and *TLR2* (encoding Toll-like receptor 2), which are highly expressed by macrophages in human atherosclerotic plaques, and *NOD2* (encoding nucleotide-binding oligomerization domain-containing protein 2), which is regulated by cytokine and TLR signaling, and is dependent on the activation of the p38 MAPK signaling pathway^24^. Several chemokines and chemokine receptor genes were also upregulated, indicating proinflammatory and migratory transcriptional reprogramming induced by atherosclerotic plasma consistent with the secretory chemokines released (Extended Data Fig. 4a,b). *IL6* was coexpressed with several *CXCL* genes, and with *NAMPT* (encoding nicotinamide phosphoribosyltransferase), which is expressed at high levels in PBMCs from patients with acute coronary syndrome and by inflammatory M1 macrophages^25^. Overall, this analysis revealed a transcriptional signature consistent with the intracellular signaling activation evident by CyTOF and the secretion of proatherogenic cytokines.

To pinpoint regulatory relationships between cell type-specific signaling pathways identified by CyTOF (for example, pCREB, pS6, pp38, pERK1/2, pMAPKAPK2) and gene expression, we integrated the GO-enriched gene expression data with the identified cell type and phosphoprotein activity pairs (Extended Data Fig. 4c,d). Filtering cross-correlations of mass cytometry and gene expression data identified CREB phosphorylation in conventional DCs and monocytes and S6 in monocytes as top correlates (Extended Data Fig. 4e). The analysis of DEGs against chromatin immunoprecipitation followed by sequencing (ChIP–seq) libraries (ENCODE and ChEA Consensus from ChIP–X and ChEA databases^26^) and sequence motif predictions (TRANSFAC and JASPAR position weight matrices) identified *CREB1*, *CREM* (encoding cAMP-responsive element modulator, a CREB family member^27^) and *E2F1* (encoding E2F TF 1, a TF that cooperates in the regulation of CREB signaling^28^) as the top candidate TFs explaining the expression of genes regulated in response to patient plasma (Extended Data Fig. 4f,g). Given that CREB is downstream of the phosphorylation of multiple intracellular kinases, including S6^29^, which was also activated in PBMCs by atherosclerotic plasma, these data suggest that CREB may be a key TF contributing to the transcriptional reprogramming triggered by atherosclerotic plasma in monocytes and DCs.

### CCL5 is a major contributor of the effect of atheroplasma

**Fig. 5:**
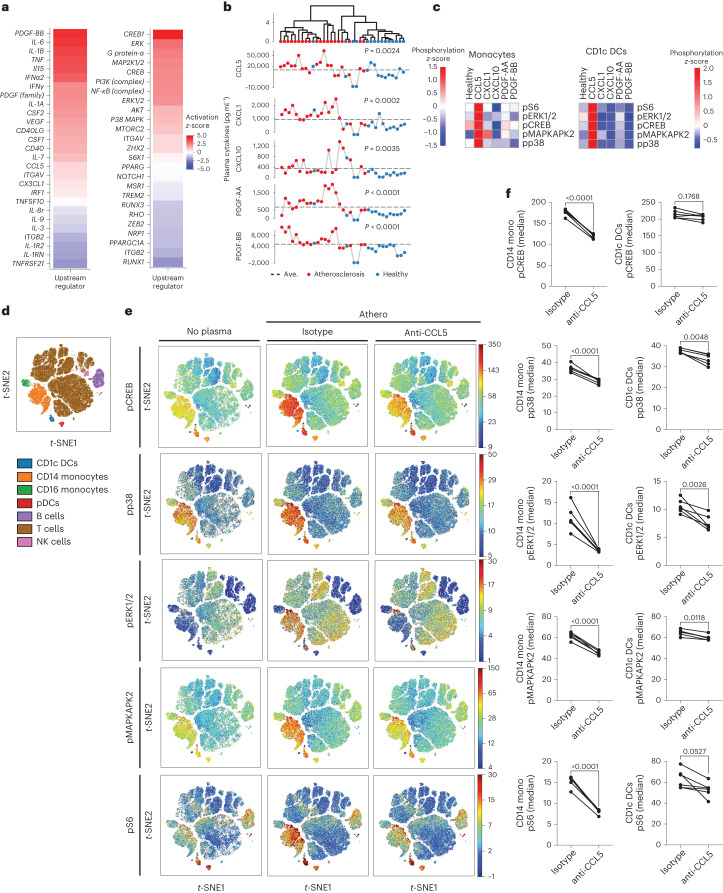
CCL5 emerges as an upstream regulator of PBMC activation upon plasma stimulation. **a**, Ingenuity Pathway Analysis performed using the DEGs in PBMCs from healthy donors stimulated with either atherosclerotic plasma (*n* = 20 biologically independent samples, 10 men) or plasma from healthy donors (*n* = 12 biologically independent samples) revealed the top upstream regulators. Upstream regulators were plotted using the activation *z*-score from Ingenuity Pathway Analysis. Fisher’s exact test was used; *P* < 0.05 was considered statistically significant. **b**, Differentially expressed cytokines in plasma from patients with atherosclerosis (*n* = 20 biologically independent samples, 10 men) versus healthy donors (*n* = 15 biologically independent samples). Dots represent plasma levels of the tested cytokines and are expressed as picograms per milliliter. Dotted line represents the average (Ave.). *P* values were calculated by two-tailed unpaired t-test; *P* < 0.05 was considered statistically significant. **c**, Heat map of phosphoprotein expression in both monocytes and CD1c^+^ DCs in PBMCs from healthy donors stimulated with healthy plasma alone (healthy) or in combination with CCL5 (10,000 pg µl^−1^), CXCL1 (600 pg µl^−1^), CXCL10 (200 pg µl^−1^), PDGF-AA (600 pg µl^−1^) and PDGF-BB (500 pg µl^−1^). *z*-Scores were used to identify the significant changes in phosphorylation levels (*n* = 3 biologically independent samples per condition). **d**, viSNE plot of all major PBMC cell types defined based on canonical expression patterns. **e**, Intracellular signaling patterns were visualized across this immune map in response to plasma from patients with atherosclerosis admixed with either an antihuman CCL5 antibody (0.16 µg µl^−1^; *n* = 6 biologically independent samples, 3 men) or isotype control antibody (0.16 µg µl^−1^; *n* = 6 biologically independent samples, 3 men). Unstimulated PBMCs were included as control (no plasma). **f**, Dot plots show the effect of CCL5 blocking on the phosphorylation of intracellular kinases in CD14^+^ monocytes and CD1c^+^ DCs after stimulation with atherosclerotic plasma. *P* values were determined by unpaired two-tailed *t*-test.

**Extended Data Fig. 5:**
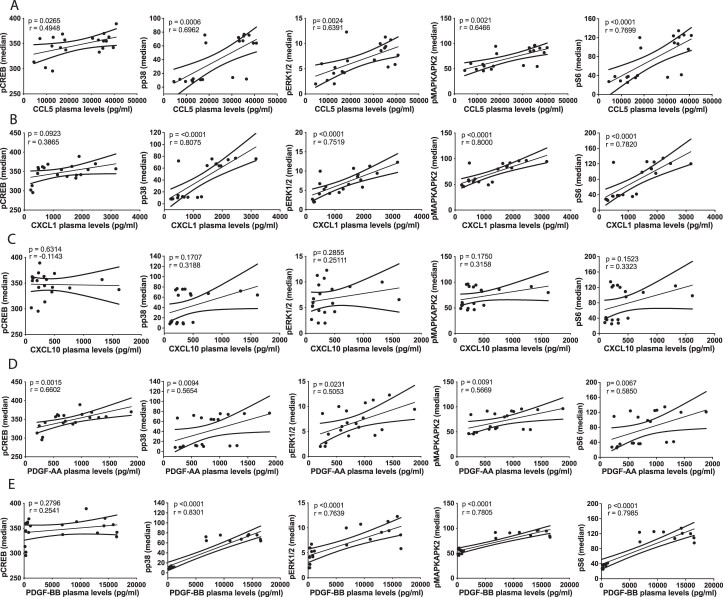
Circulating levels of candidate cytokines correlated with phospho-sites activated in monocytes. **A**. Spearman correlation of CCL5 levels in plasma of atherosclerotic patients with intracellular signal intensity of pCREB, pp38, pERK1/2, pMAPKAPK2 and pS6 phosphorylation in monocytes. **B**. Spearman correlation of CXCL1 levels in plasma of atherosclerotic patients with intracellular signal intensity of pCREB, pp38, pERK1/2, pMAPKAPK2 and pS6 phosphorylation in monocytes. **C**. Spearman correlation of CXCL10 levels in plasma of atherosclerotic patients with intracellular signal intensity of pCREB, pp38, pERK1/2, pMAPKAPK2 and pS6 phosphorylation in monocytes. **D**. Spearman correlation of PDGFAA levels in plasma of atherosclerotic patients with intracellular signal intensity of pCREB, pp38, pERK1/2, pMAPKAPK2 and pS6 phosphorylation in monocytes. **E**. Spearman correlation of PDGFBB levels in plasma of atherosclerotic patients with intracellular signal intensity of pCREB, pp38, pERK1/2, pMAPKAPK2 and pS6 phosphorylation in monocytes. P < 0.05 was considered significant. Data are from n = 20; biologically independent samples; males=10. Data are presented as mean and error with 95% CI.

To identify candidate stimuli and ligands that are responsible for PBMC activation in response to atheroplasma, we applied Ingenuity Upstream Regulator Analysis in Ingenuity Pathway Analysis to our DEG signature. This approach pinpointed upstream regulator cytokines, TFs and kinases responsible for the transcriptional signature induced by atheroplasma in PBMCs (Fig. 5a). Predicted kinase and TF genes identified included *ERK1/2, PI3K, AKT* (encoding the RAC[Rho family]-ɑ serine/threonine-protein kinase or AKT serine/threonine kinase]*, MAP2K1/2* (encoding MAP kinases 1 and 2), *p38* (encoding p38 kinase), *S6K1* (encoding S6 kinase) and *CREB*, all of which encode for proteins that were significantly phosphorylated in CD14^+^ monocytes and CD1c^+^ DCs stimulated with atheroplasma versus healthy plasma using phospho-CyTOF. Candidate cytokines included several molecules implicated in atherosclerosis (Fig. 5a), some of which were present at higher levels in atheroplasma versus healthy plasma in our analysis (Fig. 5b). Based on these computational results, and given that cytokines are known to modulate immune responses in many inflammatory conditions including atherosclerosis^30^, CCL5, platelet-derived growth factor-AA (PDGF-AA), PDGF-BB, CXCL10 (also known as IP-10) and CXCL1 (Fig. 5b) were screened for their ability to mimic the cell type-specific intracellular signaling responses associated with atheroplasma identified by CyTOF, using concentrations that corresponded to the detected plasma levels in atheroplasma (Fig. 5c). CCL5 was the main cytokine able to reproduce the monocyte signaling responses to those observed in response to atherosclerotic plasma (Fig. 5c). Positive correlations between CCL5 levels in plasma and the resulting induction of S6 (Spearman correlation coefficient (*r*) = 0.7699; *P* < 0.0001), ERK1/2 (*r* = 0.6391; *P* = 0.0024) and p38 (*r* = 0.6962; *P* = 0.0006) phosphorylation in monocytes further indicates that CCL5 is a key mediator of the CD14^+^ monocyte immune response (Extended Data Fig. 5a). To directly test this hypothesis, we incubated healthy PBMCs with atheroplasma with a CCL5-blocking antibody or an isotype control (Fig. 5d,f). CCL5 inhibition significantly reduced the activation of the signaling pathways induced by atherosclerotic plasma in CD14^+^ monocytes, although the activation of some signaling pathways in CD1c^+^ DCs persisted (that is, pCREB). Notably, although not able to fully reproduce the phosphosignaling induced by atheroplasma, CXCL1 (GRO) increased pMAPKAPK2 and PDGF-AA increased pCREB in monocytes (Fig. 5c). Moreover, the circulating levels of CXCL1, PDGF-AA and PDGF-BB significantly correlated with several phosphosites activated in monocytes (Extended Data Fig. 5b–e), indicating that the inflammatory response to atheroplasma is shaped by CCL5 in concert with CXCL1, PDGF-AA and PDGF-BB.

**Extended Data Fig. 6:**
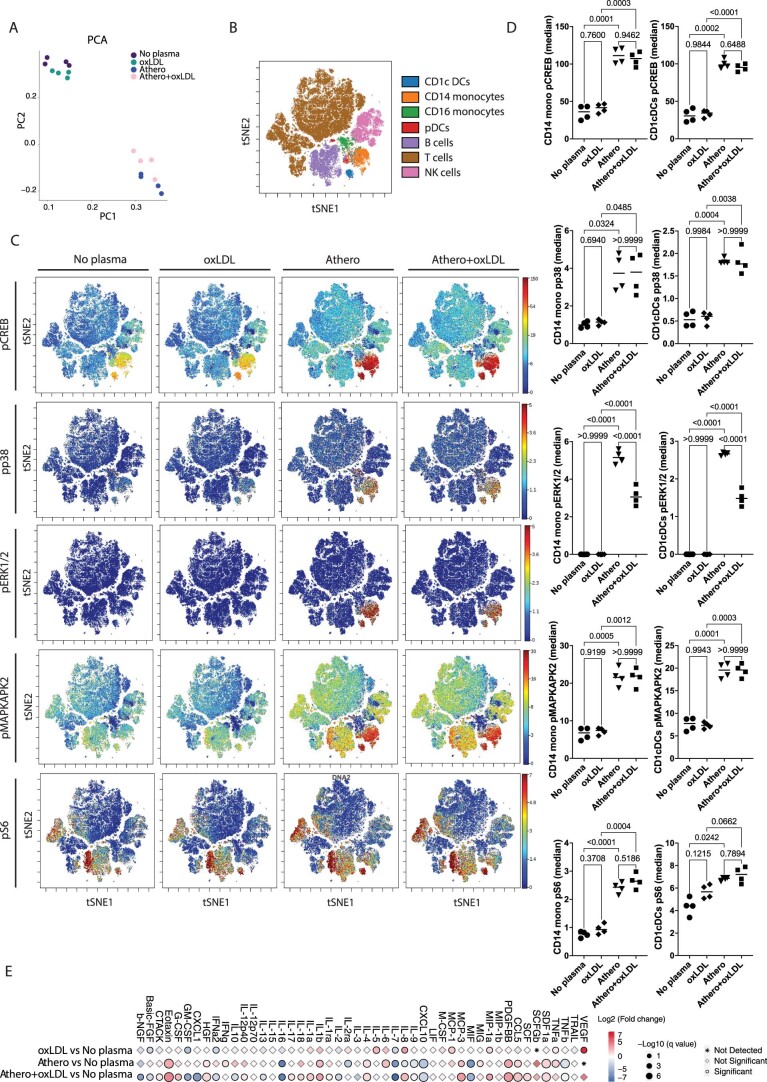
oxLDL did not reproduce the identified phosphosignaling in PBMCs stimulated with atheroplasma. **A**. Principal component analysis (PCA) of CyTOF data aggregated by cell type. **B**. viSNE plot of all major healthy PBMC cell types defined based on canonical expression patterns. **C**. Intracellular signaling patterns were visualized across this immune map in response to atheroplasma (athero) vs atheroplasma + oxLDL (athero+oxLDL), stimulation with oxLDL for 6 hours (oxLDL, 50 µg/ml) or no stimulation (no plasma). N = 4 biologically independent samples/condition; males n = 1. **D**. Dot plots show the effect of atheroplasma (athero) vs atheroplasma + oxLDL (athero+oxLDL), stimulation with oxLDL for 6 hours (oxLDL, 50 µg/ml) or no stimulation (no plasma) on the phosphorylation of intracellular kinases in CD14^+^ monocytes and CD1c^+^ DCs (n = 4/condition). P values were determined by one-way ANOVA with Tukey’s post hoc test across all groups. **E**. Point plot of released cytokines of PBMCs stimulated with atheroplasma (athero, n = 6 samples), oxLDL (n = 6 samples), atheroplasma + oxLDL (athero+oxLDL, n = 6 samples), or no stimulation (no plasma; n = 6 samples). P values were determined by paired two-tailed t-test and the BH method (FDR < 0.1) was used to correct for multiple correction.

Although all the enrolled patients presented with well-controlled lipid profiles due to statin treatment, we still investigated the possible contribution of circulating lipids to the identified phosphosignature. Circulating lipids and lipoprotein lipid content in the plasma from patients with atherosclerosis used in our phospho-CyTOF studies were quantified by nuclear magnetic resonance (NMR) spectral analysis. Pearson correlation analysis found no significant correlation between NMR lipid variables and phosphoresponse induced by atheroplasma in CD14^+^ monocytes and CD1c^+^ DCs. Significant correlations were identified between PLCG2, STAT1, STAT5 and MAPKAPK2 in CD16 monocytes, CD1c^+^ DCs and CD8^+^ T cells. Notably, these phospho-CyTOF cell pairs were not the same ones identified as significantly affected by stimulation with atheroplasma (Supplementary Table 2). Experimentally, oxidized low-density lipoprotein (oxLDL) did not reproduce the identified phosphosignaling in response to atheroplasma (Extended Data Fig. 6 and Supplementary Data Figs. 3–4). PCA of the phospho-CyTOF results confirmed that intracellular phosphorylation was driven by atheroplasma stimulation and not by oxLDL (Extended Data Fig. 6a). We found no detectable effect of oxLDL on the phosphorylation of target kinases and TFs in either CD14^+^ monocytes, CD16^+^ monocytes or CD1c^+^ DCs, indicating the identified phosphosignature was not driven by oxLDL (Extended Data Fig. 6b–d and Supplementary Figs. 3–4). Consistently, cytokines released by PBMCs after oxLDL stimulation did not recapitulate the cytokine signature seen in PBMCs stimulated with atheroplasma (Extended Data Fig. 6e). These data demonstrate that the effect of atheroplasma was not induced by oxLDL, but largely triggered by circulating CCL5, in concert with CXCL1 (GRO), PDGF-AA and PDGF-BB, leading to the activation of ERK1/2, MAP2K1/2, PI3K, CREB, p38 and S6 signaling.

### Computational prediction of drugs for atherosclerosis

Although CCL5 was identified as a major contributor to the inflammatory response induced by plasma with atherosclerosis and, as such, it could be directly targeted, inhibition of CCL5 did not fully reverse the response in CD1c^+^ DCs (Fig. 5d,f). Other circulating cytokines, including CXCL1 (GRO), PDGF-AA and PDGF-BB probably contributed to the immune response. Moreover, the cytokines identified as key drivers of the immune responses to atheroplasma in our study may not be applicable to patients with ASCVD at different stages of atherosclerosis disease and with different clinical manifestations.

To overcome patient variability in cytokine expression, we implemented a computational drug repurposing approach adaptable to the variability in immune responses from different groups of patients, with the immediate goal of discovering existing small molecules from the L1000 screening library of compounds^31,32^ predicted to reverse the inflammatory response to atherosclerotic plasma.

**Fig. 6:**
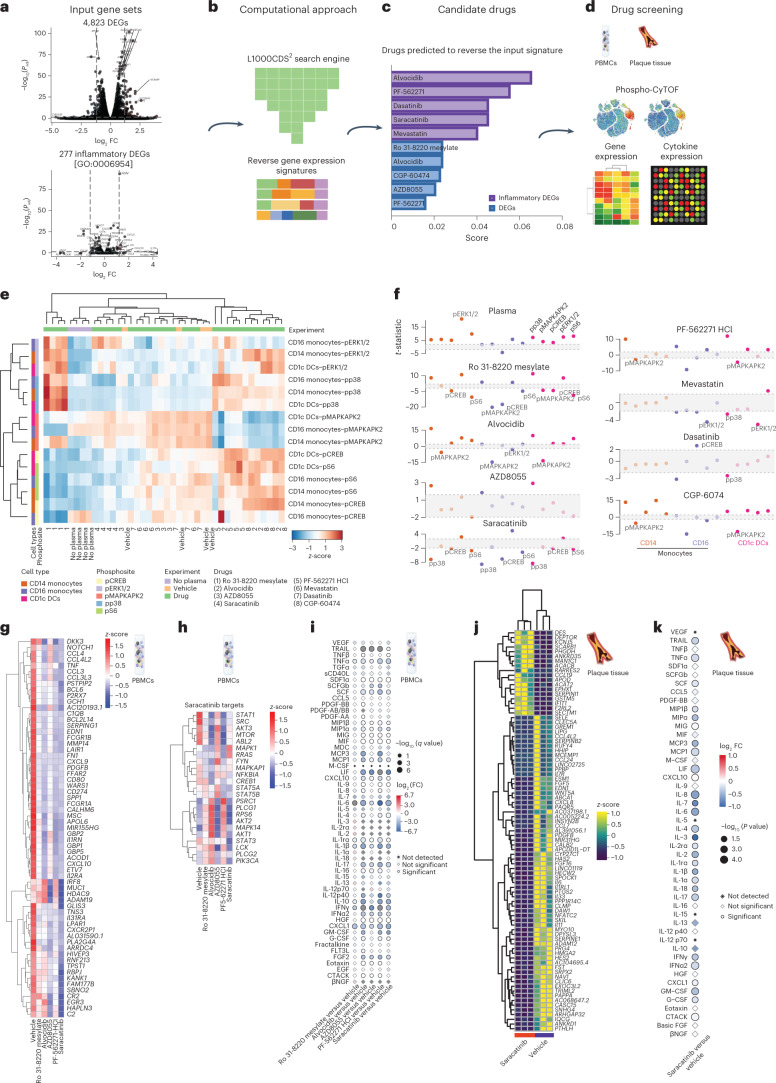
Drug repurposing computational pipeline to identify candidate anti-inflammatory small molecules for atherosclerotic disease and ex vivo screening approach. **a**, Input gene set signatures consisting of 4,823 DEGs and 277 inflammatory (GO:0006954) DEGs in healthy PBMCs, in response to atherosclerotic plasma. **b**, LINCS L1000CDS^2^ search engine used to identify drugs predicted to reverse the input transcriptional signatures. This figure was created with Biorender.com. **c**, Candidate drugs predicted to reverse the two gene set input signatures in healthy PBMCs, in response to atherosclerotic plasma. **d**, Drug screening was based on the integrated analysis of phospho-CyTOF screens, gene expression analysis and cytokine secretion by PBMCs and plaques in response to atherosclerotic plasma in the presence or the absence of candidate drugs in healthy PBMCs, in response to atherosclerotic plasma. **e**, Single-cell phosphorylation measured by CyTOF in CD1c^+^ DCs and CD14^+^ and CD16^+^ monocytes from PBMCs stimulated with atherosclerotic plasma alone (plasma) or in combination with individual top candidate small molecules (1–8), *n* = 4 biologically independent samples/condition. **f**, *t*-Statistics of monocyte- and DC-specific phosphorylation, with positive values indicating upregulation and negative values downregulation. Plasma response is compared with no plasma treatment. Each small molecule combined with plasma treatment is compared with plasma treatment alone. Points outside the gray box indicate significance (*P* < 0.05, d.f. = 6), *n* = 4 biologically independent samples per condition (2 men). **g**, Heat map of DEGs showing the *z*-scores of transcripts with absolute log_2_ FC > 0 and *q* value (adjusted p value) <0.05 in PBMCs in response to candidate drugs plus atherosclerotic plasma versus atherosclerotic plasma alone (vehicle), *n* = 4 biologically independent samples per condition (2 men). **h**, Heat map of saracatinib target and phosphosignaling genes showing the *z*-scores of transcripts in PBMCs in response to candidate drugs plus atherosclerotic plasma versus atherosclerotic plasma alone (vehicle), *n* = 4 biologically independent samples/condition (2 men). **i**, Point plot of PBMC cytokines secreted in response to candidate drugs plus atherosclerotic plasma (*n* = 4 biologically independent samples per condition, 2 men) versus atherosclerotic plasma alone (vehicle, *n* = 4 biologically independent samples per condition, 2 men). *P* values were determined by paired two-tailed *t*-test. Adjusted *P* values were considered significant. The Benjamini–Hochberg method was used to correct for multiple correction (FDR < 0.05). **j**, Heat map of DEGs showing the z-scores of transcripts with absolute log_2_ FC > 1.2 and *q* value <0.001 in atherosclerotic tissue in response to saracatinib plus atherosclerotic plasma (saracatinib) versus atherosclerotic plasma alone (vehicle), *n* = 3 samples per condition. **k**, Point plot of cytokines secreted by atherosclerotic tissue in response to saracatinib plus atherosclerotic plasma (saracatinib) versus atherosclerotic plasma alone (vehicle), *n* = 3 biological samples per condition. *P* values were determined by unpaired *t*-test. Illustrations of representative heat maps in **d** and representative PBMC tubes and arteries in **g**–**k** were created in Biorender.com.

**Extended Data Fig. 7:**
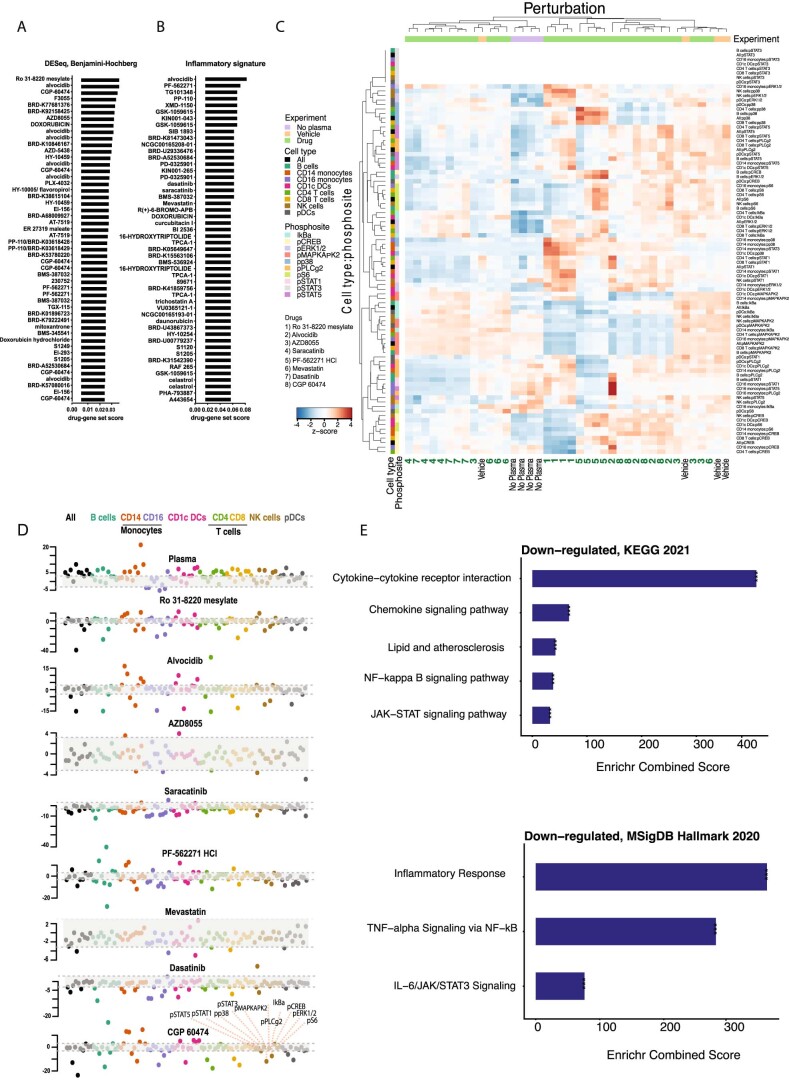
Small molecules screening identified saracatinib as negative regulator of inflammatory signatures in PBMCs and atherosclerotic tissue. **A**. Top 50 candidate small molecules predicted using 4,823 DEGs input gene set in LINCS-L1000CDS2 in healthy PBMCs, in response to atherosclerotic plasma. **B**. Top 50 candidate small molecules predicted using 277 inflammatory (GO:0006954) DEGs as gene set input in LINCS-L1000CDS^2^. **C**. Heatmap of phosphosite responses to selected candidate drugs across all immune cell types summarizing the signaling responses to atherosclerotic plasma alone (vehicle) or in combination with candidate small-molecules (1-8), n = 4 biologically independent samples/condition; males=2. **D**. t-statistics of cell-type-specific phosphorylation of all immune cells with positive values indicating up-regulation and negative values down-regulation. Plasma response is compared to no plasma treatment. Each small-molecule combined with plasma treatment is compared to plasma treatment alone. Points outside the grey box indicate significance (p < 0.05, df=6), n = 4/condition. **E**. Signaling pathway analysis of the DEGs downregulated by saracatinib in atherosclerotic tissue treated with atherosclerotic plasma alone (vehicle) or in combination with saracatinib. The combined score (*c*) was calculated from the *p* value (*p)* obtained using Fisher’s exact test. P < 0.05 was considered significant.

First, the identified transcriptional signatures of 4,823 DEGs and the subnetwork of 277 DEGs associated with inflammatory response (GO:0006954) were compared to the large-scale gene expression Library of Integrated Network-Based Cellular Signatures (LINCS) L1000 data using the L1000CDS^2^ tool^31^. The L1000CDS^2^ search engine comprises 389,031 perturbation experiments, covering 62 cell lines and 3,924 small molecules, calculated from the LINCS L1000 dataset^32^ using the characteristic direction signature method^33^ (Fig. 6a,b). With this method, the therapeutic prediction assumes that a small molecule that exerts an opposing effect (reverse mode) on the gene expression signature to that observed in PBMCs stimulated with atherosclerotic plasma would interfere with and potentially reverse the inflammatory response. A ranked candidate list of small molecules predicted to reverse the input transcriptional signature of atherosclerotic plasma stimulation of PBMCs for each comparison was produced (Fig. 6c and Extended Data Fig. 7a,b). The highest scoring small molecules from the input signature of 4,823 DEGs included Ro 31-8220 mesylate, a PKC inhibitor; alvocidib (also listed as F3055 or HY-1005/flavoropirol), a flavonoid alkaloid and potent cyclin-dependent kinase (CDK) inhibitor, a CDK9 kinase inhibitor and a CDC25 phosphatase family inhibitor; CGP-60474, also a CDK inhibitor; and the mTOR inhibitor AZD8055 (Fig. 6c). Among the small molecules identified using the 277 genes associated with inflammatory response (GO:0006954) were PF-562271, a FAK inhibitor; dasatinib, an inhibitor of the SRC family of protein tyrosine kinases; and saracatinib (AZ0530), a dual inhibitor of the tyrosine kinases c-SRC and ABL (half maximal inhibitory concentration (IC_50_) of 2.7 and 30 nM, respectively), Fyn (IC_50_ of 10 nM), and other tyrosine kinases c-YES, LYN, BLK, FGR and LCK (IC_50_ from 4 to 10 nM). Although not a top hit, the first discovered statin mevastatin^34^ was also identified and included in further validation efforts (Fig. 6c). Doxorubicin was identified using both input gene sets (Extended Data Fig. 7a,b) but excluded from further analysis because of its low score and known cardiotoxicity^35^.

### Phospho-CyTOF screening of selected candidate drugs

Next, we examined the effect of the top eight candidates (Fig. 6c) on cell type-specific phosphorylation and gene and cytokine expression directly in human samples (Fig. 6d and Extended Data Fig. 7c,d). Ro 31-8220 mesylate and saracatinib induced a significant inhibition of most of the specific kinase and TF phosphorylation induced by atherosclerosis plasma (Extended Data Fig. 7c,d), with cell specificity for monocytes and CD1c^+^ DCs (Fig. 6e,f). Specifically, Ro 31-8220 mesylate reduced the phosphorylation of CREB and S6 in CD14^+^ and CD16^+^ monocytes and in CD1c^+^ DCs, and reduced the phosphorylation of MAPKAPK2 in CD16^+^ monocytes and CD1c^+^ DCs. Unlike other tested compounds, saracatinib treatment significantly inhibited the phosphorylation of most of the tested kinases and TFs, including p38, CREB and S6 in CD14^+^ and CD16^+^ monocytes and in CD1c^+^ DCs. Mevastatin was included in the screening because it was a statin computationally predicted to reverse the inflammatory signature induced by atheroplasma (Fig. 6c). Mevastatin had no major effect on the targets activated by atherosclerotic plasma (for example, CREB and S6), but did reduce the phosphorylation of ERK1/2 in CD16^+^ monocytes and CD1c^+^ DCs and of p38 in CD1c^+^ DCs. The absence of a broad effect of mevastatin on immune cell signaling further supports the need to identify new immunotherapeutics to reduce the residual chronic inflammation in atherosclerosis.

### Effect of candidate drugs on immune genes and cytokines

**Extended Data Fig. 8:**
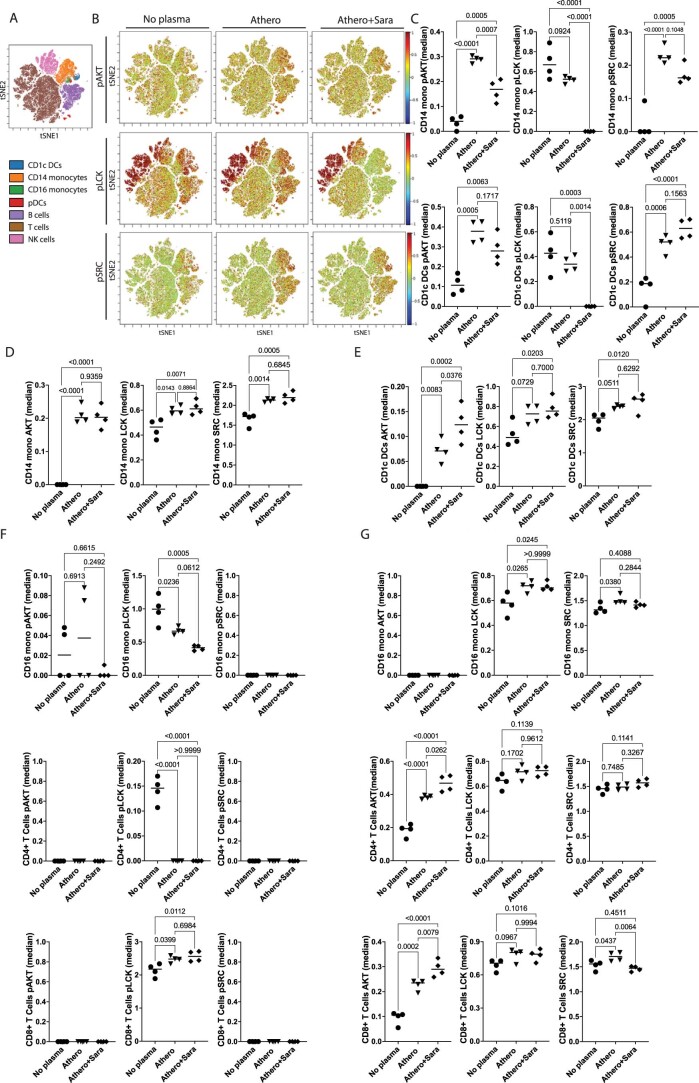
Saracatinib altered the signaling induced by athero plasma by reducing the activation of SRC, LCK and PI3K/Akt/mTOR signaling. **A**. viSNE plot of major cell types in healthy PBMC defined based on canonical expression patterns. **B**. The phosphorylation signaling of AKT, LCK, SRC was visualized across this immune map in response to atheroplasma (athero) vs atheroplasma + saracatinib 10 µM (athero+sara), or no stimulation (no plasma); n = 4 biologically independent samples/condition, males=1. **C**. Dot plots show the effect of atheroplasma (athero) vs atheroplasma + saracatinib 10 µM (athero+sara), or no stimulation (no plasma) on the phosphorylation of AKT, LCK, SRC in CD14^+^ monocytes and CD1c^+^ DCs, n = 4 biologically independent samples/condition, males=1. P values were determined by one-way ANOVA with Tukey’s post hoc test across all groups. **D**. Dot plots show the effect of atheroplasma (athero) vs atheroplasma + saracatinib 10 µM (athero+sara), or no stimulation (no plasma) on protein levels of AKT, LCK, SRC in CD14^+^ monocytes; n = 4 biologically independent samples/condition, males=1. P values were determined by one-way ANOVA with Tukey’s post hoc test across all groups. **E**. Dot plots show the effect of atheroplasma (athero) vs atheroplasma + saracatinib 10 µM (athero+sara), or no stimulation (no plasma) on total protein levels of AKT, LCK, SRC in CD1c^+^ DCs; n = 4 biologically independent samples/condition, males=1. P values were determined by one-way ANOVA with Tukey’s post hoc test across all groups. **F**. Dot plots show the effect of atheroplasma (athero) vs atherosplasma + saracatinib 10 µM (athero+sara), or no stimulation (no plasma) on phosphorylation and total protein levels of AKT, LCK, SRC in CD16+ monocytes, CD4 + T cells, CD8 + T cells; n = 4 biologically independent samples/condition, males=1. P values were determined by one-way ANOVA with Tukey’s post hoc test across all groups. **G**. Dot plots show the effect of atheroplasma (athero) vs atheroplasma + saracatinib 10 µM (athero+sara), or no stimulation (no plasma) on total protein levels of AKT, LCK, SRC in CD16+ monocytes, CD4 + T cells, CD8 + T cells; n = 4 biologically independent samples/condition, males=1. P values were determined by one-way ANOVA with Tukey’s post hoc test across all groups.

To further establish the predicted anti-inflammatory efficacy of the candidate drugs, we tested their ability to reverse the transcriptional signature and release of inflammatory and proatherogenic cytokines induced by atherosclerotic plasma in PBMCs (Fig. 6g–i). Consistent with the computational prediction, all tested drugs reduced the expression of most genes upregulated by atherosclerotic plasma. However, the effect of saracatinib appeared stronger and more specific to reducing the identified inflammatory signature (Fig. 6g,h). Saracatinib specifically reduced *CREB1* (encoding CREB), *AKT3* and *MTOR* (encoding AKT and mTOR that are part of the PI3K/AKT/mTOR signaling pathway), in addition to reducing the saracatinib targets *SRC (*encoding SRC proto-oncogene, nonreceptor tyrosine kinase), *ABL2* (encoding ABL proto-oncogene 2, nonreceptor tyrosine kinase) and *LCK* (encoding lymphocyte-specific protein tyrosine kinase) (Fig. 6h). To further elucidate how saracatinib alters the signaling induced by atheroplasma, we performed additional phospho-CyTOF experiments to study LCK, SRC and AKT. We confirmed that atheroplasma increased the protein expression of SRC and AKT and their phosphorylation in CD14^+^ monocytes. Notably, saracatinib significantly reduced the phosphorylation of AKT and LCK. The phosphorylation of SRC was also reduced, but not significantly (Extended Data Fig. 8). The anti-inflammatory effect of saracatinib was further confirmed by the reduced secretion of several proatherogenic cytokines such as IL-6 and IL-1β, GM-CSF, G-CSF, CXCL1, CXCL10 and IFNγ (Fig. 6i). This effect was shared with that of the other drugs, with Ro 31-8220 mesylate being the least effective at reducing cytokine secretion in response to atheroplasma (Fig. 6i).

Based on the combined ability of saracatinib to reverse the atherosclerotic plasma-induced phosphorylation of kinases and TFs, transcriptional signature and cytokine expression and release, we tested its effect directly on human atherosclerotic plaque tissue ex vivo. Saracatinib treatment of cultured plaque tissue downregulated the expression of several genes encoding inflammatory cytokines and genes involved in cytokine–cytokine receptor interactions, chemokine signaling and inflammatory pathways, such as IL-6/JAK/STAT3 and NF-κB signaling (Fig. 6j and Extended Data Fig. 7e). The anti-inflammatory effect of saracatinib was further confirmed by the parallel reduction of secreted cytokines from atherosclerotic plaques (Fig. 6k), an effect largely overlapping to that seen in PBMCs.

Saracatinib-treated plaques upregulated *DEPTOR*, encoding the EP domain-containing mTOR-interacting protein (DEPTOR), an inhibitor of mTORC1/2 signaling, indicating negative regulation of AKT/mTOR signaling. Upregulated genes in saracatinib-treated plaques also included *SCARB1*, which regulates macrophage cholesterol homeostasis and reverse cholesterol transport, and *APOD*, a component of high-density lipoprotein (HDL) that is involved in the transient interaction between HDL and LDL and in lipid metabolism. Other upregulated genes were *ACACB*, a mitochondrial gene involved in fatty acid metabolism, and *ACAT2*, which regulates the esterification of cholesterol in plaque macrophages (Fig. 6j). Overall, these results suggest that saracatinib not only exerts anti-inflammatory and antiatherosclerotic effects on circulating immune cells but also directly on human atherosclerotic tissue.

### Effect of saracatinib on PBMCs from patients with carotid or coronary disease

Based on these promising results, and due to its oral availability and safety profile in clinical trials^36,37^, we selected the phase 2a-ready compound saracatinib for further investigation. The IC_50_ of CREB, S6, p38 and MAPKAPK2 in human monocytes ranged from 0.8 to 28 µM (Supplementary Fig. 5a). Next, we used our established CyTOF screening strategy to compare the anti-inflammatory effect of saracatinib on PBMCs isolated from patients with carotid artery disease (carotid endarterectomy (CEA) versus CAD, and then incubated the cells with either autologous plasma or healthy donor plasma. There was no significant difference in the inhibitory effect of saracatinib on major intracellular phophosites in CD14^+^ monocytes and DCs from patients with CEA or CAD (Supplementary Fig. 5b), suggesting that saracatinib is equally effective regardless of the vascular site affected by atherosclerotic disease.

### Saracatinib inhibits atherosclerosis progression

**Fig. 7:**
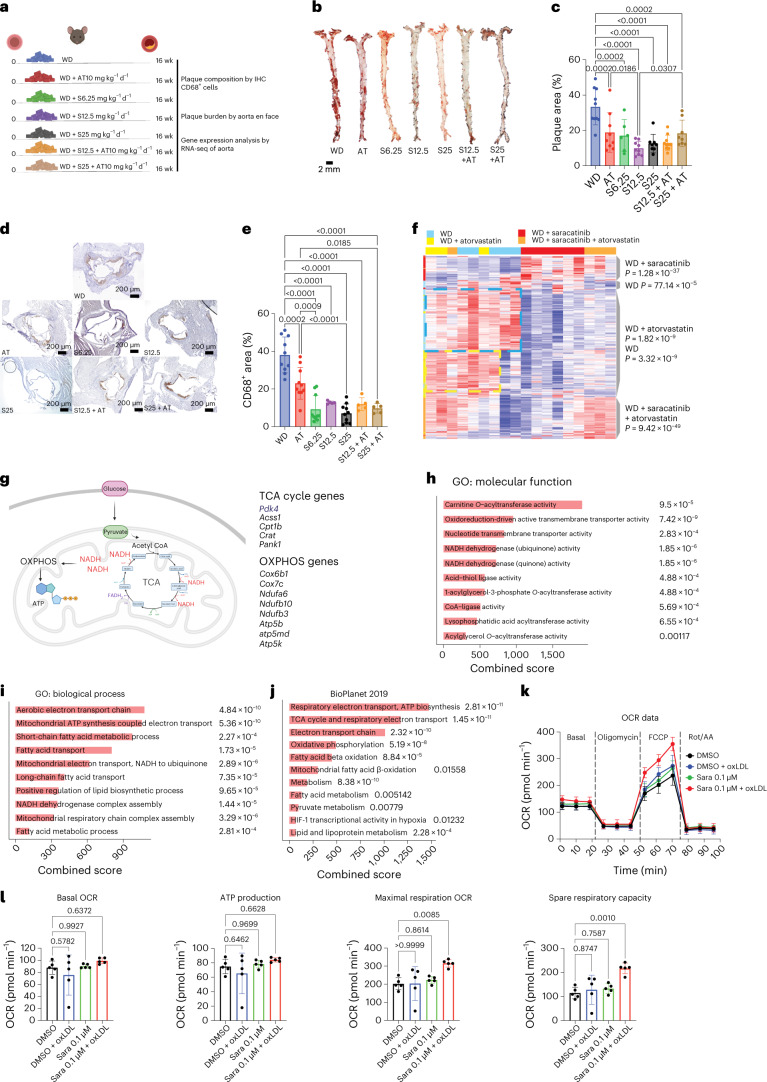
Effect of saracatinib on atherosclerosis in vivo. **a**, Experimental design to study the effect of saracatinib 6.25 (S6.25), 12.5 (S12.5) or 25 (S25) mg kg^−1^ d^−1^; atorvastatin (AT; 10 mg kg^−1^ d^−1^); or the combination of saracatinib and atorvastatin (S + AT; 12.5 or 25 and 10 mg kg^−1^ d^−1^) admixed to WD on plaque burden, composition and gene expression compared with WD alone in male *Apoe*^*−/−*^ mice. This figure was created with Biorender.com. **b**, Representative images of en face preparation of aortas stained with ORO. **c**, Bar graphs with overlapping dots of en face ORO^+^ area quantification (plaque area). WD, *n* = 10; AT, *n* = 9; S6.25, *n* = 6; S12.5, *n* = 9; S25, *n* = 8; S12.5 + AT, *n* = 10; S25 + AT, *n* = 8 mice. *P* values were determined by one-way ANOVA with Tukey’s post hoc test across all groups. **d**, Representative images of CD68 immunostaining of the aortic root (4× magnification). **e**, Bar graphs with overlapping dots of CD68^+^ area quantification. WD, *n* = 10; AT, *n* = 10; S6.25, *n* = 10; S12.5, *n* = 5; S25, *n* = 10; S12.5 + AT, *n* = 5; S25 + AT, *n* = 5 mice. *P* values were determined by one-way ANOVA with Tukey’s post hoc test. Data are presented as mean ± s.d. **f**, Heat map of the top 100 DEGs hierarchically clustered expressed in the aortas across treated mice. WD, *n* = 5; AT, *n* = 3; S, *n* = 6; S + AT, *n* = 4 mice. Rows, *z*-scored gene expression values; columns, individual aortas. The treatment categories are indicated above the heat map and the dendrograms on the right indicate the DEGs enriched in the different categories. *P* values were determined by the two-sided binomial proportions test. **g**, Top DEGs involved in the TCA cycle and OXPHOS induced by saracatinib in the aortas of treated mice, *n* = 6. This figure was created with Biorender.com. **h**, GO molecular functions of the top 100 DEGs upregulated in the saracatinib group, *n* = 6 mice. The combined score (c) was calculated from the *P* value obtained using Fisher’s exact test. **i**, GO Biological Process of the top 100 DEGs upregulated in the saracatinib group, n = 6 mice. The combined score was calculated from the *P* value obtained using Fisher’s exact test. **j**, BioPlanet signaling pathway analysis of the top 100 DEGs upregulated in the saracatinib group, *n* = 6 mice. The combined score was calculated from the *P* value obtained using Fisher’s exact test. **k**, OCR and respiratory parameters in mouse BMDMs treated with vehicle (dimethylsulfoxide (DMSO), *n* = 5 biologically independent samples), DMSO + oxLDL (*n* = 4 biologically independent samples), 0.1 µM saracatinib (sara) or 0.1 µM saracatinib + oxLDL (*n* = 5 biologically independent samples/condition). Data are presented as mean ± s.d. **l**, Summary of respiratory parameters in mouse BMDMs treated with vehicle (DMSO), DMSO + oxLDL, 0.1 µM saracatinib or 0.1 µM saracatinib + oxLDL (*n* = 5, biologically independent samples per condition): basal OCR, ATP production, maximal respiratory OCR, spare respiratory capacity. *P* values were determined by one-way ANOVA with Dunnet’s post hoc test vs vehicle. Data are presented as mean ± s.d.

To determine whether saracatinib can exert antiatherosclerotic and anti-inflammatory effects and work as well as the gold-standard atorvastatin at reducing atherosclerosis progression in vivo, we fed apolipoprotein E-deficient (*Apoe*^−/−^; 6-week-old) mice a Western diet (WD) for 16 weeks with or without various dosages of saracatinib and/or atorvastatin admixed into their food (Fig. 7). In brief, *Apoe*^−/−^ mice were randomized into the following treatment groups: (1) WD only; (2) WD containing atorvastatin (10 mg kg^−1^ d^−1^), which was selected as a standard-of-care control; (3) WD containing saracatinib (6.25 mg kg^−1^ d^−1^); (4) WD containing saracatinib (12.5 mg kg^−1^ d^−1^); (5) WD containing saracatinib (25 mg kg^−1^ d^−1^); (6) WD containing atorvastatin and saracatinib (10 and 12.5 mg kg^−1^ d^−1^, respectively); and (7) WD containing atorvastatin and saracatinib (10 and 25 mg kg^−1^ d^−1^) (Fig. 7a). The selected dosages of saracatinib corresponded to therapeutically viable ranges established in phase 1 and phase 2 clinical trials^36,37^. Specifically, the mouse dosage of 6.25 mg kg^−1^ d^−1^ was equivalent to 30 mg d^−1^ in humans, 12.5 mg kg^−1^ d^−1^ translated into 60 mg d^−1^ and the dosage of 25 mg kg^−1^ d^−1^ into 125 mg d^−1^ (ref. ^38^).

**Extended Data Fig. 9:**
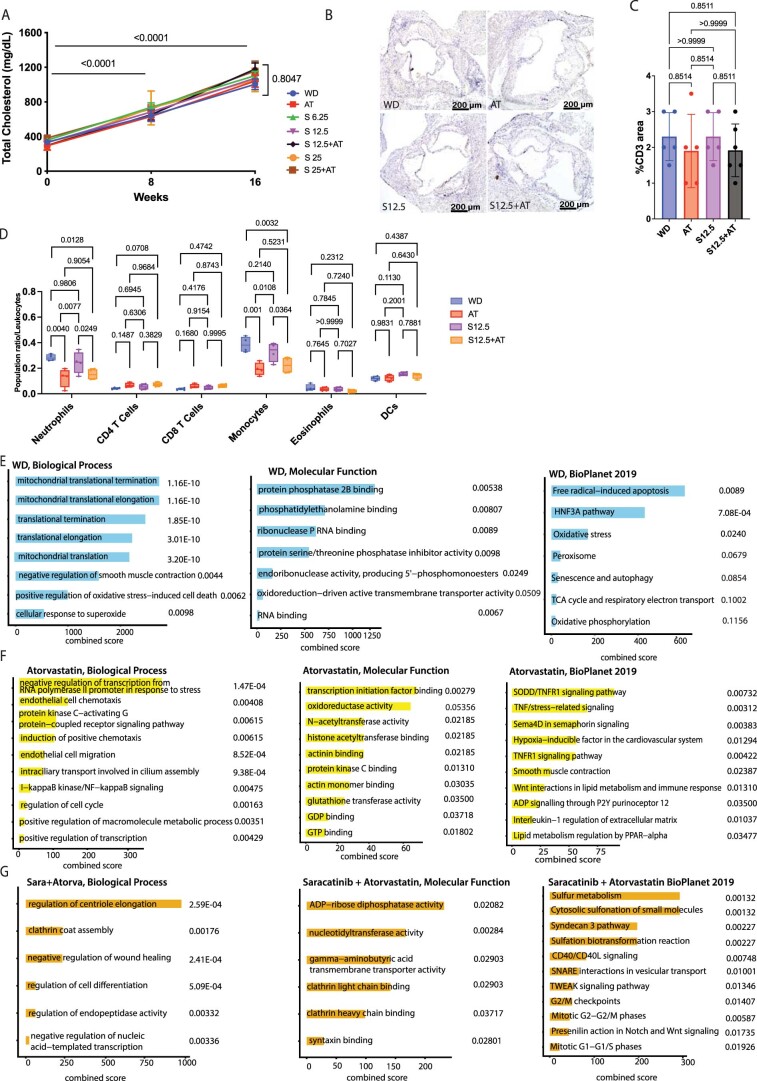
Effect of saracatinib on both circulating mouse immune cell and plaque lesions. **A**. Total cholesterol levels in plasma of ApoE-/- mice treated with western diet (WD), WD plus atorvastatin (10 mg/kg/day) (AT), WD plus saracatinib 6.25 mg/kg/day (S6.25), WD plus saracatinib 12.5 mg/kg/day (S12.5), WD plus saracatinib 25 mg/kg/day (S25), WD plus atorvastatin (10 mg/kg/day) and saracatinib 12.5 mg/kg/day (S12.5 + AT), WD plus atorvastatin (10 mg/kg/day) and saracatinib 25 mg/kg/day (S25 + AT); WD: n = 10; AT: n = 10; S6.25: n = 10; S12.5: n = 10; S25: n = 10; S12.5 + AT: n = 4; S25 + AT: n = 4 mice. Two-way ANOVA with Dunnett post-hoc correction for multiple comparisons. Data are presented as mean values +/- SEM. **B**. Representative images of CD3 immunostaining of the mouse aortic root done across the following treatments: WD, AT, S 12.5 and AT + S 12.5. (4X magnification). **C**. Bar graphs with overlapping dots of CD3^+^ area quantification. P values were determined by one-way ANOVA with Tukey’s post hoc test across all groups. WD: n = 5; AT: n = 5; S12.5: n = 5; S12.5 + AT: n = 6 mice. Data are presented as mean values +/- SD. **D**. CyTOF analysis of circulating immune cell from available whole blood from mice samples at 16 weeks. Major immune cell populations in blood (neutrophils, CD4 T cells, CD8 T cells, monocytes, eosinophils and dendritic cells (DCs) were analyzed across the following treatments: WD, AT, S 12.5 and AT + S 12.5. Box plots and whiskers showing the mean and range (min to max). P values were determined by one-way ANOVA with Tukey’s post hoc test across all groups. n = 4 mice/group. **E**. GO Molecular functions and signaling pathway analysis of the top 100 DEGs upregulated in the WD group. (n = 5 mice) The combined score (c) was calculated from the p-value obtained using Fisher’s exact test. **F**. GO Molecular functions and signaling pathway analysis of the top 100 DEGs upregulated in the atorvastatin group, (n = 3 mice). The combined score (c) was calculated from the p-value obtained using Fisher’s exact test. **G**. GO Molecular functions and signaling pathway analysis of the top 100 DEGs upregulated in the saracatinib+atorvastatin group, n = 4 mice. The combined score (c) was calculated from the p-value obtained using Fisher’s exact test.

Atorvastatin reduced plaque burden by 40% compared with WD, an effect that was not associated with a reduction of total cholesterol plasma levels (Fig. 7b,c and Extended Data Fig. 9a). As expected, saracatinib did not affect total cholesterol plasma levels, either alone or when used in combination with atorvastatin (Extended Data Fig. 9a). Consistent with our computational predictions, saracatinib reduced plaque burden by ~46% when used at the lowest dosage (6.25 mg kg^−1^ d^−1^), by ~68% at the intermediate dosage of 12.5 mg kg^−1^ d^−1^ and reached a plateau with ~60% reduction at the highest dosage (25 mg kg^−1^ d^−1^) (Fig. 7b,c). A significantly greater reduction in plaque burden was induced by 12.5 mg kg^−1^ d^−1^ saracatinib versus atorvastatin. The effect of saracatinib on plaque burden was accompanied by a significant decrease in plaque macrophage content, measured by CD68 staining of aortic root cross-sections, versus WD (Fig. 7d,e). The effects of 6.25 and 25 mg kg^−1^ d^−1^ saracatinib and 25 mg kg^−1^ d^−1^ saracatinib plus atorvastatin on plaque macrophages was superior to that of atorvastatin alone (Fig. 7d,e). The analysis of CD3^+^ T cells in the atherosclerotic lesions showed no difference across groups, suggesting a specific effect of saracatinib on macrophages (Extended Data Fig. 9b,c). Saracatinib alone did not significantly affect circulating levels of immune cells, indicating a plaque-specific therapeutic effect. In fact, changes in circulating levels of neutrophils and monocytes were observed only in combination with atorvastatin, and this effect was similar to that achieved with atorvastatin alone and was probably unrelated to saracatinib (Extended Data Fig. 9d).

To understand how saracatinib alters gene expression in atherosclerotic plaques, we analyzed RNA-seq data from the atherosclerotic aorta of treated mice (Fig. 7f–j). As expected, WD treatment induced upregulation of genes involved in mitochondrial dysfunction and oxidative stress signaling (Extended Data Fig. 9e), whereas genes upregulated by atorvastatin treatment were implicated in oxidoreductase activity, smooth muscle cell contraction, actin binding, chemotaxis and NF-κB signaling, which suggests a residual inflammatory signature in the atherosclerotic aorta of mice treated with statins (Extended Data Fig. 9f). Hierarchical clustering of the top 100 DEGs across treatments identified two distinct modules of upregulated genes enriched in the aortas of mice treated with saracatinib (51 genes) and saracatinib plus atorvastatin (84 genes) (Fig. 7f and Extended Data Fig. 9g). A third module consisted of a shared signature of 219 upregulated genes between the WD and atorvastatin groups that could be further clustered into two distinct submodules. One consisted of 111 upregulated genes specific to the WD condition that are involved in epithelial-to-mesenchymal transition (*P* < 0.0009; MSigDB Hallmark 2020), and an atorvastatin module (*P* = 2.07 × 10^−28^) that included genes involved in smooth muscle contraction (*P* = 0.0066; BioPlanet 2019) and macrophage inflammatory signaling (*P* = 0.03; PhenGenI Association 2021). The other submodule was associated with atorvastatin treatment alone and included 77 upregulated genes, including those involved in TNF and TNF receptor 1 (TNFR1) signaling (*P* < 0.005; BioPlanet 2019), TGFβ signaling pathway (*P* = 0.04; Panther 2016), NF-κB signaling (*P* = 0.001; Elsevier Pathway collection) and chemoattractant activity (*P* = 0.007; GO:0042056).

Saracatinib treatment induced the expression of a set of 51 genes involved in the metabolism of glucose-derived pyruvate through the tricarboxylic acid (TCA) cycle and mitochondrial oxidative phosphorylation (OXPHOS), which convert pyruvate to acetyl coenzyme A (acetyl-CoA)^39^ (Fig. 7f–j). This transcriptional metabolic reprogramming indicates a skewing toward anti-inflammatory and proresolving macrophages^39^, and is consistent with the antiatherogenic effects of saracatinib on plaque size and composition in mice. Saracatinib, specifically upregulated *Acss1* (*acyl-CoA synthetase short chain family member 1*), encoding a mitochondrial acetyl-CoA synthase enzyme that catalyzes the conversion of acetate to acetyl-CoA; *Cpt1b*, encoding carnitine palmitoyltransferase 1B, which is required for the net transport of long-chain fatty acyl-CoAs from the cytoplasm to the mitochondria; *Crat*, encoding carnitine acetyltransferase, which catalyzes the reversible transfer of acyl groups and regulates the ratio of acyl-CoA:CoA; and *Acsl1*, encoding acyl-CoA synthetase long chain family member 1, which converts free long-chain fatty acids into fatty acyl-CoA esters^40^. *Pank1*, encoding pantothenate kinase 1, a key regulatory enzyme in the biosynthesis of CoA, was also expressed in the aorta of saracatinib-treated mice. Upregulated genes involved in OXPHOS were *Cox6b1* and *Cox7c*, encoding subunits of the terminal enzyme of the mitochondrial respiratory chain, and a series of genes encoding proteins involved in mitochondrial OXPHOS and NADH dehydrogenation (that is, *Ndufa6*, *Ndufb10* and *Ndufb3*) and adenosine 5′-triphosphate (ATP) synthesis (that is, *Atp5b*, *Atp5md* and *Atp5k*)^41^. Of note, saracatinib also increased the expression of *Pdk4*, encoding pyruvate dehydrogenase kinase 4, which regulates glucose metabolism^42^, and *Prkar2b*, encoding protein kinase cAMP-dependent type II regulatory subunit β, which interacts and suppresses the transcriptional activity of CREB^43^. This effect is consistent with our observations by CyTOF that CREB is activated by atherosclerotic plasma in macrophages and DCs, and that CREB phosphorylation is inhibited by saracatinib.

To confirm the impact of saracatinib treatment on the electron transport chain and OXPHOS pathways identified by the RNA-seq analysis from the atherosclerotic aorta of mice, we designed an experiment where bone marrow-derived macrophages (BMDMs) were incubated with oxLDL to reproduce the proinflammatory environment found within the atherosclerotic plaque^44^. First, we performed a dose–response curve experiment (0.1–10 µM) and selected the concentration of 0.1 µM because it showed no impact on baseline mitochondrial respiration (basal oxygen consumption rate (ORC) and ATP production) in BMDMs either with or without oxLDL (Supplementary Fig. 5c,d). Next, BMDMs were coincubated with oxLDL (50 µg ml^−1^) and saracatinib (0.1 µM) for 6 h and assessed for mitochondrial metabolism using the extracellular flux assay (Fig. 7k,l). Saracatinib, which confirmed no effect on basal OCR or ATP production of oxLDL-treated BMDMs, induced significantly higher electron transport chain activity, measured as the maximal respiration OCR induced by the uncoupler carbonyl cyanide 4-(trifluoromethoxy) phenylhydrazone (FCCP) compared to controls (Fig. 7l). Furthermore, saracatinib significantly increased spare respiratory capacity (Fig. 7l). Treatments with either oxLDL or saracatinib alone showed no effect, indicating that the beneficial effect of saracatinib was specific to BMDMs exposed to oxLDL, an effect relevant to the lipid-rich plaque microenvironment. The increase in mitochondrial fitness induced by saracatinib in oxLDL-treated BMDMs is consistent with the transcriptional metabolic reprogramming induced by saracatinib in atherosclerotic aortas, suggesting that metabolic reprogramming probably contributes to the antiatherogenic effect of saracatinib.

### Effects of saracatinib in a rabbit model of atherosclerosis

**Fig. 8:**
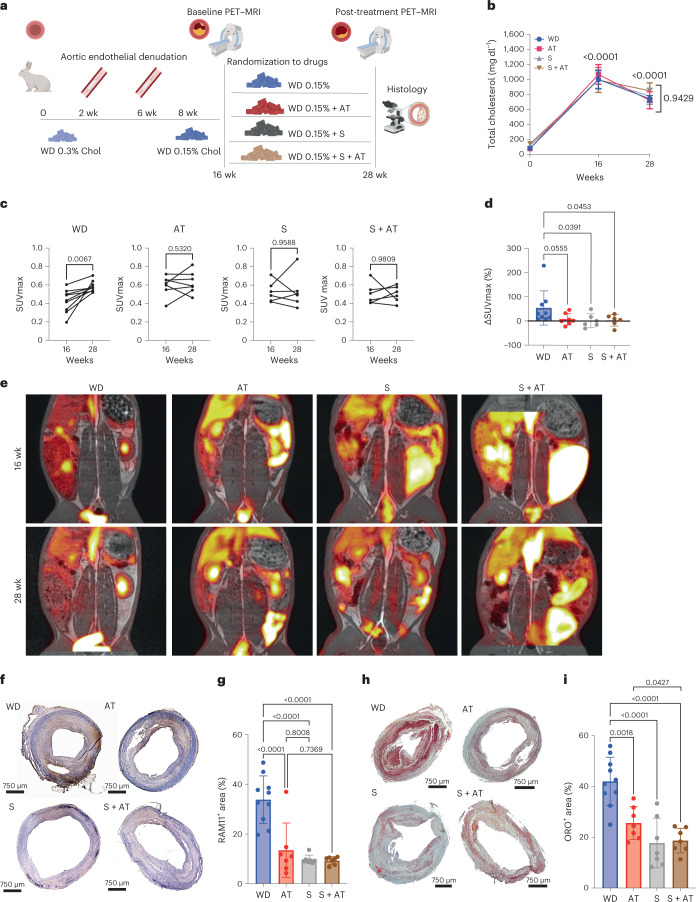
Effect of saracatinib in a rabbit model of advanced atherosclerosis. **a**, Experimental design to study the effect of 4 mg kg^−1^ d^−1^ saracatinib (S), 3 mg kg^−1^ d^−1^ atorvastatin (AT) or the combination of saracatinib and atorvastatin (S + AT; at 4 and 3 mg kg^−1^ d^−1^) on existing plaques in male New Zealand white rabbits. This figure was created with Biorender.com. **b**, Total cholesterol levels in plasma of rabbits treated with WD, WD plus AT, S and S + AT. Two-way ANOVA with Tukey’s post hoc correction for multiple comparisons. Data are presented as mean ± s.e.m. WD, *n* = 10; AT, *n* = 6; S, *n* = 8; S + AT, *n* = 8 rabbits. **c**, Pretreatment (16 weeks) and post-treatment (28 weeks) [^18^F]FDG uptake by the atherosclerotic arterial wall measured as SUVmax in each treatment group. WD, *n* = 9; AT, *n* = 7; S, *n* = 6; S + AT, *n* = 6. *P* values were determined by paired two-tailed t-test. **d**, Changes in [^18^F]FDG uptake by the atherosclerotic arterial wall between 16 and 28 weeks, measured as change in SUVmax (ΔSUVmax) in each treatment group. WD, *n* = 9; AT, *n* = 7; S, *n* = 6; S + AT, *n* = 6. One-way ANOVA with Fisher’s least significant difference test versus WD. Data are presented as mean ± s.e.m. **e**, Representative images of [^18^F]FDG PET–MRI of rabbit aortas for each group at 16 and 28 weeks. **f**, Representative images of RAM11 immunostaining of the rabbit abdominal aortas (4× magnification). **g**, Bar graphs with overlapping dots of RAM11^+^ area quantification. WD, *n* = 10; AT, *n* = 7; S, *n* = 7; S + AT, *n* = 7 rabbits. *P* values were determined by one-way ANOVA with Tukey’s post hoc test across all groups. Data are presented as mean ± s.d. **h**, Representative images of ORO staining of the rabbit abdominal aortas (4× magnification). **i**, Bar graphs with overlapping dots of ORO^+^ area quantification. WD, *n* = 10; AT, *n* = 7; S, *n* = 7; S + AT, *n* = 7 rabbits. *P* values were determined by one-way ANOVA with Tukey’s post hoc test across all groups. Data are presented as mean ± s.d. Chol, cholesterol.

To establish whether saracatinib treatment could also be beneficial in the setting of advanced atherosclerotic plaques, we used a validated rabbit model of atherosclerosis^45–48^ (Fig. 8a). In this model, advanced lesions present pathological features that resemble those of human coronary disease, and the atherosclerotic abdominal aortas can be imaged longitudinally in vivo using noninvasive [^18^F]fluorodeoxyglucose positron emission tomography–magnetic resonance imaging ([^18^F]FDG PET–MRI), which is directly translatable to the clinic^48–50^. [^18^F]FDG uptake by the arterial wall correlates with plaque macrophage content and metabolic activity in both atherosclerotic rabbits and patients, and with circulating biomarkers of atherosclerotic plaque inflammation and clinical cardiovascular risk factors or cardiovascular risk scores^51^. Briefly, atherosclerosis was induced in New Zealand white rabbits by a combination of a WD with 0.3% cholesterol and two balloon endothelial denudations^46,48^. At 8 weeks of the 16-week atherosclerosis induction period, the diet was switched to 0.15% cholesterol. At 16 weeks, all animals underwent a baseline [^18^F]FDG PET–MRI and were immediately randomized into the following groups: (1) WD only; (2) WD containing atorvastatin (3 mg kg^−1^ d^−1^); (3) WD containing saracatinib (4 mg kg^−1^ d^−1^); and (4) WD containing saracatinib and atorvastatin (4 and 3 mg kg^−1^ d^−1^, respectively). All groups were imaged again at 28 weeks, 3 months after treatment initiation. The selected dosage of saracatinib corresponded to 25 mg kg^−1^ d^−1^ in mice and 125 mg d^−1^ in humans^38^. As expected, plasma levels of total cholesterol were significantly increased due to the 0.3% cholesterol WD during the induction period, and were similarly reduced in all groups at 28 weeks due to the switch to 0.15% cholesterol WD (Fig. 8b).

**Extended Data Fig. 10:**
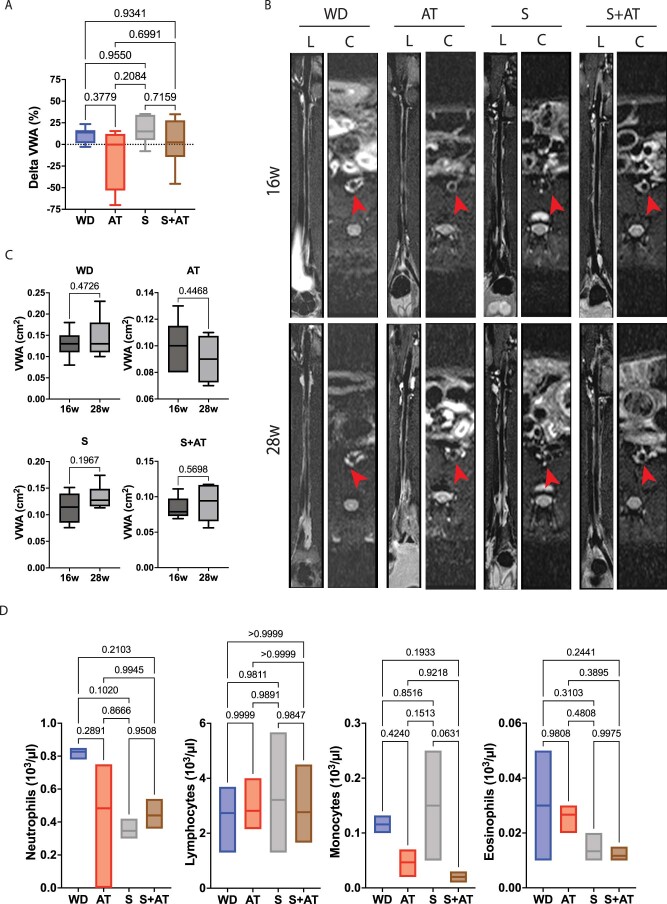
Effect of Saracatinib in a Rabbit Model of Advanced Atherosclerosis. **A**. Changes in vessel wall area (VWA, cm^2^) between 16 weeks and 28 weeks measured as the percentage of the difference between outer and inner wall area (delta VWA %) in each treatment group, WD: n = 7; AT: n = 4; S12.5: n = 6; S12.5 + AT: n = 6. One-way ANOVA with Tukey’s post hoc test across all groups. Box plots showing the mean and range (min to max). **B**. Representative cross-sectional MRI images of rabbit aortas for each group at 16 and 28 weeks (L=longitudinal sections; C=cortical sections) Red arrows indicate the aorta position. **C**. Pre-treatment (16 weeks) and post-treatment (28 weeks) vessel wall area (VWA, cm^2^) in each treatment group. P values were determined by unpaired two-tailed t-test. **D**. Bar graphs of complete blood count (CBC) for each group measuring the concentration of neutrophils, lymphocytes, monocytes and eosinophils (10^3^/ul) at 28 weeks, n = 3 rabbits/group. Floating bars showing the mean and range (min to max). P values were determined by one-way ANOVA with Tukey’s post hoc test across all groups.

[^18^F]FDG uptake by the atherosclerotic arterial wall at 28 weeks, measured as maximum standardized uptake value (SUVmax), was significantly increased compared with baseline (16 week) in WD rabbits (Fig. 8c–e). Atorvastatin induced a stabilization of [^18^F]FDG uptake in the arterial wall, indicating reduced progression of plaque inflammation (Fig. 8c). This result was expected and in agreement with a previous [^18^F]FDG imaging study where the same dose and duration of atorvastatin treatment was used to treat rabbits with atherosclerosis^52^. Similarly, saracatinib-treated rabbits showed no increase in SUVmax at the end of treatment, indicating reduced progression of plaque inflammation. A similar effect was achieved with saracatinib plus atorvastatin treatment (Fig. 8c). The analysis of changes in SUVmax between baseline and after treatments highlighted that saracatinib reduced the progression of plaque inflammation versus WD (Fig. 8d,e). The anti-inflammatory effect of saracatinib in combination with atorvastatin was similar to that observed when saracatinib was used alone, indicating no additive effect of the drug when used with a statin under our experimental conditions. No difference in the MRI vessel wall area was detected in the WD groups, and before and after treatment across groups (Extended Data Fig. 10a–c). Histological analysis of the atherosclerotic arterial wall showed that atorvastatin significantly reduced macrophage content in plaques, measured as RAM11 staining of abdominal aorta cross-sections (Fig. 8f,g). The effect of saracatinib on plaque macrophages, alone or in combination with atorvastatin, was similar to that of atorvastatin (Fig. 8f,g). Oil Red O (ORO) staining revealed a significant reduction of lipid content in plaques in all treatment groups compared with WD controls. Notably, a significant reduction was observed in rabbits treated with the combination of saracatinib and atorvastatin versus atorvastatin alone (Fig. 8h,i). Meanwhile, circulating levels of immune cells were not significantly affected by saracatinib, atorvastatin or their combination (Extended Data Fig. 10d).

## Discussion

Although inflammation contributes to ASCVD beyond dyslipidemia and high cholesterol, lipid-lowering therapy remains the sole pharmacologic standard of care to reduce the risk of ischemic events in patients with ASCVD^4^. To date, the inflammatory component of the disease remains untreated^5^. To help bridge this gap, we developed a systems immunology-based drug repurposing approach that leverages high-dimensional and single-cell modeling of the immunological alterations in samples of patients with ASCVD. Using phospho-CyTOF, we discovered that atherosclerotic plasma drives single-cell phosphorylation patterns of intracellular kinases and TFs specifically activated in circulating monocytes and DCs, and identified corresponding transcriptional signatures and cytokine expression patterns. Circulating immune cells from healthy patients or those with ASCVD responded similarly to atheroplasma, indicating that circulating factors in patients with atherosclerosis were responsible for the identified transcriptional and phosphosignatures that result in monocyte and DC activation. Our study also highlighted important intrinsic differences between patient and healthy immune cells. Counterintuitively, unstimulated PBMCs from patients with atherosclerosis had lower baseline phosphorylation for most intracellular proteins versus healthy donors, probably as a result of previous exposure to autologous atherosclerotic plasma. However, as healthy donors were not matched for age, the influence of age difference cannot be excluded. Despite this lower baseline phosphosignature, CD14^+^ monocytes and CD1c^+^ DCs from both patients and healthy donors showed very similar activation in response to atherosclerotic plasma. However, when exposed to healthy plasma, only immune cells from patients were activated, suggesting they have an intrinsic inflammatory capacity in response to the same stimulus. The analysis of the transcriptional signature and the activation of intracellular transduction cascades induced by atheroplasma revealed the activation of SRC and LCK signaling, PI3K/AKT/mTOR signaling, MAPK/p38 signaling and the downstream activation of CREB and S6 in CD14^+^ monocytes and CD1c^+^ DCs. These signals ultimately result in the secretion of several proinflammatory and proatherogenic cytokines. Mechanistically, we identified CCL5 as a major contributor to the innate immune response to atheroplasma, and found that this response was unrelated to the fact that patients treated with statins had well-controlled lipid levels. Yet, when CCL5 was directly targeted, its inhibition showed residual activation of DCs, indicating that CCL5 probably acts in concert with other circulating cytokines (for example, CXCL1, PDGF-AA and PDGF-BB). Moreover, there may be additional effectors of the immune responses besides cytokines.

To address this, we applied a computational approach, using our disease transcriptional signatures as input, to identify drugs predicted to reverse the inflammatory response. Those drugs were then screened using the same single-cell phospho-CyTOF and multiomics approach directly in cells from patients with atherosclerosis. Saracatinib had a stronger and more specific inhibition of the identified signature than the other compounds. For example, the activation of phosphosites in CD14^+^ and CD1c^+^ DCs activated by atheroplasma was significantly reduced in healthy PBMCs after saracatinib treatment. Saracatinib also reduced the expression of several genes upregulated by atheroplasma, including transcripts encoding proteins involved in the identified phosphosignature (that is, *CREB1*, *AKT3* and *MTOR*) and *SRC*, *ABL2* and *LCK*, encoding established saracatinib targets. Using an ex vivo model of human atherosclerotic plaques exposed to atherosclerotic plasma, we confirmed the anti-inflammatory effects of saracatinib directly, as shown by the downregulation of genes encoding inflammatory cytokines, chemokine signaling and other inflammatory pathways, including IL-6/JAK/STAT3 and NF-κB signaling. Saracatinib treatment of human atherosclerotic vascular explants upregulated *DEPTOR*, encoding an inhibitor of mTORC1/2 signaling, indicating a specific negative regulation of the PI3K/AKT/mTOR signaling activated by atheroplasma. Saracatinib treatment of human plaques also upregulated genes involved in cholesterol metabolism and reverse cholesterol transport, the pathway that removes cholesterol from lipid-rich plaque macrophages, further suggesting antiatherogenic properties^39^ beyond the regulation of plaque inflammation.

Importantly, the antiatherosclerotic efficacy of saracatinib was tested in both a mouse and rabbit model of atherosclerosis. In mice, saracatinib effectively reduced plaque burden and plaque inflammation, an effect that was superior to that of atorvastatin. This effect was associated with transcriptional metabolic reprogramming in the artery wall that included the expression of genes involved in the TCA cycle and mitochondrial OXPHOS. This effect is consistent with the saracatinib-mediated inhibition of SRC and PI3K/mTOR activation, signaling pathways known to impair mitochondrial electron transport chain complexes involved in OXPHOS and the TCA cycle^53,54^. Metabolic reprogramming after saracatinib treatment was confirmed in oxLDL-treated macrophages and was consistent with the metabolic state of proresolving macrophages. In a rabbit model of atherosclerosis that generates complex human-like atherosclerotic plaques^52,55,56^, saracatinib also had an anti-inflammatory effect on atherosclerotic lesions, as shown by the stabilization of [^18^F]FDG uptake in the arterial wall and reduced plaque macrophage content at histology. [^18^F]FDG uptake correlates significantly and consistently with plaque macrophage content, and its reduction indicates reduced progression of plaque inflammation in both rabbits and patients^50,57^. The anti-inflammatory effect of saracatinib was similar to that of atorvastatin. Plaque lipid content was also reduced, a result that aligns with the transcriptional TCA cycle and mitochondrial OXPHOS signature observed in the atherosclerotic aorta of mice and the upregulation of genes involved in reverse cholesterol transport in human atherosclerotic vascular explants treated with saracatinib. Our in vivo studies were designed to test the hypothesis that saracatinib had an effect similar to that of atorvastatin in halting atherosclerosis inflammation progression versus placebo (WD control or progression). By acting through different pathways, saracatinib has the potential to be used as an anti-inflammatory treatment for patients who are already treated with lipid-lowering drugs. This hypothesis will need to be tested in future phase 2 clinical studies.

In conclusion, our systems immunology-driven drug repurposing framework led to the identification of a use for an existing drug to aid the development of targeted immunotherapies for ASCVD. We show that scalable and cost-effective single-cell technologies, such as CyTOF for ex vivo phospho-CyTOF screenings directly in human samples from patients, offer a suitable platform to select candidate drugs for further screening into preclinical studies before moving into early-phase clinical trial planning.

## Methods

### Subject cohort

Thirty-four patients with ASCVD undergoing either CEA (*n* = 26) or coronary angiogram (CAD; *n* = 8) at the Mount Sinai Medical Center were enrolled in a clinical study approved by the Institutional Review Board (IRB) of the Icahn School of Medicine at Mount Sinai (IRB no. 11-01427) and the IRB of the New York University (NYU) Langone Health (IRB no. i21-00429). Twenty-four healthy donors were also enrolled. Eligible subjects gave informed, written consent to participate in the study. No compensation was provided. Exclusion criteria were current infection, autoimmune disease, active or recurrent cancer, severe renal failure requiring dialysis and peripheral arterial occlusive disease with rest pain. Healthy donors’ exclusion criteria were age <18 years, dyslipidemia, high blood pressure, diabetes and history of cardiovascular disease. The average age for the healthy donors was 60 ± 6 years old (median, 61 years), 46% were men, 54% were women, 54% were White, 4.2% were Black, 4.2% were Asian, 4.2% were unknown and 33.4% did not report their race. Supplementary Table 1 summarizes the demographic and clinical characteristics of patients. Plasma and PBMCs isolated from patients and healthy donors were used for functional ex vivo studies using phospho-CyTOF, RNA-seq and cytokine expression analyses.

### PBMC stimulation and phospho-CyTOF analysis

Fasting peripheral venous blood was collected preoperatively in the presence of Anticoagulant Citrate Dextrose Solution A (BD Vacutainer, BD364606) to isolate plasma and PBMCs, as described in ref. ^18^. Stimulation of PBMCs and staining protocols for CyTOF were based on methods described by ref. ^58^. Briefly, patient PBMCs (2 × 10^6^ cells ml^−1^ in RPMI-1640 medium, 1% FBS, 1× Penicillin/Streptomycin (P/S)) were stimulated with either autologous plasma or control plasma from healthy donors at a final concentration of 20% for 15 min. Healthy PBMCs were stimulated with either autologous healthy plasma or patient plasma (final concentration 20%) for 15 min. For the oxLDL experiments, PBMCs were preincubated with oxLDL for 3 h for cytokine experiment and 6 h for Phospho-CyTOF experiments (50 µg ml^−1^; catalog no. L34357; Thermo Fisher Scientific) and stimulated with patient plasma (20%) for 3 h for the cytokine studies and 15 min for the phosphosignaling experiments. For the cytokine screening experiment, healthy PBMCs were stimulated for 15 min with autologous plasma (20%) and the following cytokines: CCL5 (10,000 pg µl^−1^; catalog no. 580206; BioLegend), CXCL1 (600 pg µl^−1;^ catalog no. 574406; BioLegend), CXCL10 (200 pg µl^−1^; catalog no. 573506; BioLegend), PDGF-AA (600 pg µl^−1;^ catalog no. PHG0035; Life Technologies) and PDGF-BB (500 pg µl^−1;^ catalog no. 577306; Biolegend). Cytokine concentrations were within the concentrations measured in plasma from patients. For the CCL5 blocking antibody experiment, healthy PBMCs were stimulated for 15 min with patient plasma preincubated (15 min) with either isotype control antibody (0.16 µg µl^−1;^ catalog no. MAB002; R&D Systems) or antihuman CCL5 antibody (0.16 µg µl^−1^; catalog no. MAB2781; R&D Systems). Unstimulated PBMCs were included as control. For the phospho-CyTOF screening of candidate small molecules, PBMCs were preincubated with each drug for 30 min as follows: drug 1, Ro 31‐8220 mesylate (10 µM; catalog no. S7207; Selleckchem,); drug 2, alvocidib (1 µM; catalog no. S1230; Selleckchem); drug 3, AZD8055 (10 µM; catalog no. S1555; Selleckchem); drug 4, saracatinib (10 µM; catalog no. S1006; Selleckchem); drug 5, PF‐562271 HCl (10 µM; catalog no. S7357; Selleckchem); drug 6, mevastatin (10 µM; catalog no. S4223; Selleckchem); drug 7, dasatinib (0.1 µM; catalog no. SYN-1036; SYNkinase), drug 8, CGP-60474 (0.37 µM; catalog no. 5471; TOCRIS Bioscience). PBMCs were then stimulated with patient plasma (final concentration 20%) for 15 min. For all experiments, the Rh103 nucleic acid intercalator (0.125 nM; Fluidigm) was added as a viability marker, and signal transduction was stopped by fixing the PBMCs with formaldehyde (final concentration 1.6%; Thermo Scientific) for 10 min at RT. PBMC samples were barcoded using Cell-ID 20-Plex Pd Barcoding Kit (Fluidigm) and labeled with a panel of cell surface antibodies to identify major immune subsets, and then permeabilized with ice-cold methanol before staining with a cocktail of antibodies against intracellular phosphoprotein epitopes (Supplementary Table 3). Samples were washed and stored in freshly diluted 2% formaldehyde (Electron Microscopy Sciences) in PBS containing 0.125 nM 191-iridium nucleic acid intercalator until acquisition using a CyTOF2 Helios mass cytometer (Standard BioTools–FKA Fluidigm) at an event rate of <400 events per second. Data were normalized using the Helios normalizer software (Fluidgm), barcoded samples were deconvoluted and doublets were filtered as described in ref. ^18^. No statistical methods were used to predetermine sample size, but our sample sizes are similar to those reported in previous publications^18,58^.

### CyTOF analysis

CyTOF data analysis and visualizations were performed using Cytobank (Beckman Coulter Life Sciences). Phosphosignaling experiments were first analyzed using viSNE^59^ on cell surface markers, and expression of the phosphoproteins was added as a third parameter on the viSNE map to compare relative expression across immune subsets. The median phosphosite abundances of gated CyTOF data were compared before and after plasma treatment using *t*-tests for each combination of gated cell type and phosphosite parameters. Similarly, we tested the effect of drugs on plasma-treated cells using *t*-tests assuming identical d.f. for the purposes of comparing effects of drug perturbations between combinations of cell types and phosphosites. To visualize comparisons, *t*-statistic values were plotted, stratified by cell type and phosphosites. A gray box was used to indicate significant values outside of the box.

### Luminex multiplex assay

Cytokines in culture supernatant and in plasma were measured using a human cytokine/chemokine magnetic bead panel (MILLIPLEX MAP, HCYTMAG-60K-PX41 and the Bio-Rad plate Human Cytokine Screening Panel 48-Plex Kit) and a Luminex 200 multiplex immunoassay system (Luminex Corporation) per the manufacturers’ instructions. Data were analyzed using either the Milliplex Analyst 5.1 software (EMD Millipore) or Xponent software (Luminex Corporation). Cytokine release from PBMCs was calculated by normalizing each value in the supernatant for the concentration of each cytokine in corresponding plasma as follows: net release (pg ml^−1^) = supernatant concentration (pg ml^−1^) − 20% plasma concentration (pg ml^−1^). Data were log-transformed and unpaired, two-sided *t*-tests were used for each comparison. Subsequently, *P* values were adjusted using Benjamini–Hochberg correction. *P* values less than 0.05 were considered statistically significant. The fold change (FC) between each comparison was calculated and log_2_-transformed and cytokines with log_2_ FC > 0 were defined as upregulated, and cytokines with log_2_ FC < 0 were defined as downregulated. Statistical analysis was performed in R (v.4.0.3). Spearman correlation analysis identified significant correlations (*P* < 0.05) between plasma levels of candidate cytokines and phosphoprotein levels.

### Heat maps of CyTOF and Luminex data

Standardized data (*z*-scores) were calculated for each feature. Heat maps were rendered in R (v.4.0.3) and ordered based on hierarchical clustering with Euclidian distance metric and complete linkage, unless otherwise specified. Batch corrections were applied to aggregate mass cytometry and cytokine data using empirical Bayes batch correction (Combat)^60^.

### PBMC RNA-seq and analysis

Total RNA extracted from PBMCs using the RNeasy Mini Kit (Qiagen) was used for poly(A) library preparation and sequencing (Illumina HiSeq 2500 and Novaseq 6000 SP 100 Cycle Flow Cell v.1.5; Illumina). Spliced Transcripts Alignment to a Reference (STAR)^61^ was used to map reads to the human genome (GRCh38; Gencode v.24). Transcript counts were analyzed using the DESeq R package^62^. For the RNA-seq PCA, log pseudo-counts (*n* + 1) normalized by size factors were used. DEGs were called with an adjusted *P* value threshold of 0.05, corresponding to an FDR of 5%. This analysis yielded 4,823 DEGs. To determine DEGs specific to inflammation, significant DEGs were filtered for the GO term inflammatory response (GO:0006954), yielding 227 DEGs.

### Gene set enrichment analysis

DEGs were submitted to Enrichr^23,63^. Enriched TFs supported by ChIP–seq data and position weight matrix predictions^23^ were ranked based on the Enrichr combined score: the product of the negative log *P* value from the Fisher’s exact test and the *z*-score of the deviation from the expected rank. Enriched GO terms and signaling pathway enrichment for upregulated and downregulated DEGs were ranked based on the Enrichr −log_10_ values (adjusted *P* values).

### Small-molecule predictions

To predict small molecules that reverse plasma-induced transcriptional changes in PBMCs, the identified 4,823 DEGs and the 227 DEGs (GO:0006954) were submitted to L1000CDS^2^ with the reverse option^31^. The prediction score of L1000CDS^2^ is the cosine distance between input genes and DEGs after perturbation of 3,924 small molecules in 62 cell lines assessed by the LINCS L1000 platform. The prediction score quantifies the number of genes that, after drug perturbation, are upregulated when the input is downregulated, and vice versa.

### Ex vivo human atherosclerotic vascular explant screens

Atherosclerotic surgical tissue was cut into small pieces (3 × 3 × 3 mm^3^). Pieces from each portion of the plaque (for example, core and shoulder) were included for all the experimental conditions and were cultured in RPMI-1640 medium (Corning Cellgro) with autologous plasma (20%) and either saracatinib (25 µM) or vehicle for 24 h at 37 °C, 5% CO_2_. The supernatant was collected and stored at −80 °C for the Luminex multiplex assay, as described above for PBMCs. Tissue was stored in Allprotect Tissue Reagent (Qiagen) for RNA-seq analysis. Cytokine expression was measured using the Bio-Rad plate Human Cytokine Screening Panel 48-Plex Kit as per the manufacturer’s instructions on a Luminex 200 multiplex immunoassay system. Data were analyzed as described for PBMCs. Total RNA from human atherosclerotic tissue was isolated using the gentleMACS Octo Dissociator (Miltenyi Biotec) homogenization protocol using QIAzol Lysis Reagent (catalog no. 79306; Qiagen) and RNAeasy Mini Kit (catalog no. 74104; Qiagen). RNA (2 ng) was used for low-input Clontech SMART-Seq HT with Nxt HT poly(A) library construction and sequencing (Novaseq 6000 SP 100 Cycle Flow Cell v.1.5; Illumina).

### Analysis of RNA-seq data from saracatinib-treated tissue

For quality control of RNA-seq data, a data processing pipeline was implemented in Snakemake. To ensure reproducibility, a conda environment was set. Source code and the detailed steps are available on GitHub (https://github.com/mgildea87/RNAseq_PE). Quality control of RNA-seq data was performed using FastQC2 v.0.11.9, and a final report was generated for each FASTQ file to assess general sequencing quality. Raw sequenced reads were trimmed using fastp v.0.20.1 for quality of bases and to eliminate sequencing adapters. The raw reads were then aligned using STAR v.2.6.1d with hg38 as the reference genome. The gene-level expression counts were computed with the featureCounts function in the Subread package (v.1.6.3; parameters -g gene_id -Q5-s2) using the human gene annotations from Gencode release 33. The raw counts were processed using R package DESeq2 (v.1.30.1) for differential expression analysis between conditions. For hierarchical clustering analysis, raw values were size factor normalized and standardized and plotted using pheatmap package (v.1.0.12) in R. The volcano plot was created using ggplot2 (v.3.3.6). DESeq2 was used to identify DEGs using a cutoff of adjusted *P* = 0.001, a threshold of 1.2-FC, and normalized counts >4. For hierarchical clustering analysis, raw values were size factor normalized and standardized and plotted using pheatmap package (v.1.0.12) in R.

### Lipid profiling of human atherosclerotic samples

Total lipids and lipoprotein lipid content was measured using a 250-biomarker metabolic platform based on NMR spectroscopy (Nightingale Health). The 11 lipid variables included in the analysis were total lipids (total.lipids), total free cholesterol (total.FC), remnant cholesterol (remnant.C), clinical LDL cholesterol (clinical.LDL.C), LDL cholesterol (LDL.C), LDL free cholesterol (LDL.FC), HDL cholesterol (HDL.C), non-HDL cholesterol (non.HDL.C), HDL free cholesterol (HDL.FC), very-low-density lipoprotein (VLDL) free cholesterol (VLDL.FC) and VLDL cholesterol (VLDL.C). Pearson correlation analysis was used to detect significant correlations (*P* < 0.05) between lipid variables and phosphoprotein levels (Supplementary Table 2).

### Animal experiments

All animal experiments were approved by the Institutional Animal Care and Use Committees of the Icahn School of Medicine at Mount Sinai (IACUC-2016-0032) and NYU Grossmann School of Medicine (IACUC PROTO202100030).

### Mouse model of atherosclerosis

Eighty 6-week-old male *Apoe*^*−/−*^ mice (B6.129P2-Apoe^tm1Unc^/J; Jackson Laboratories) were housed at the Icahn School of Medicine at Mount Sinai (12-h light–dark cycle conditions; temperature 20–24 °C, 30–70% humidity). *Apoe*^*−/−*^ mice were randomized into seven groups and fed the following diets for 16 weeks: (1) WD (percentage kilocalories protein, 17.3%; carbohydrate, 43%; fat, 40%; catalog no. D12079B; Research Diet), (2) WD plus 10 mg kg^−1^ d^−1^ atorvastatin (catalog no. D16102901; Research Diet), (3) WD plus 6.25 mg kg^−1^ d^−1^ saracatinib (catalog no. D17112004; Research Diet), (4) WD plus 12.5 mg kg^−1^ d^−1^ saracatinib (catalog no. D16102904; Research Diet), (5) WD plus 12.5 mg kg^−1^ d^−1^ saracatinib and 10 mg kg^−1^ d^−1^ atorvastatin (catalog no. D16102905; Research Diet), (6) WD plus 25 mg kg^−1^ d^−1^ saracatinib (catalog no. D17112005; Research Diet) and (7) WD plus 25 mg kg^−1^ d^−1^ saracatinib and 10 mg kg^−1^ d^−1^ atorvastatin (catalog no. D18110802; Research Diet). Atorvastatin was purchased from Sigma-Aldrich (catalog no. PHR1422) and saracatinib was purchased from Selleckchem (catalog no. S1006). Blood was collected before randomization and at 8 weeks of treatment from the submandibular vein in lithium heparin. After 16 weeks of treatment, mice were anesthetized (isofluorane 2%) and blood was collected with cardiac puncture (1 ml) in lithium heparin. After perfusion with PBS, the heart was harvested for aortic root analysis and the aorta was harvested for either en face analysis or RNA-seq. No statistical methods were used to predetermine sample size, but our sample sizes are similar to those reported in previous publications^64,65^.

### Total cholesterol analysis

Plasma total cholesterol levels were determined using the Cholesterol E Kit (catalog no. 999-02601; FUJIFILM Wako Diagnostics). Normal distribution of data was confirmed using the Shapiro–Wilk test and Kolmogorov–Smirnov test. Statistical differences were tested using one-way analysis of variance (ANOVA) with Tukey’s post hoc test across all groups and two-way ANOVA with Dunnett’s post hoc test versus baseline (Prism v.9 for MacOS).

### CyTOF analysis of mouse whole blood

Blood collected at 16 weeks of treatment was resuspended in the Proteomic Stabilizer Prot1 buffer (catalog no. PROT1; Smart Tube) and stored at −80 °C. After thawing the samples in a 10–15 °C water bath (for ~10 min), erythrocytes were lysed twice using 1× Thaw-Lyse buffer (Smart Tube), incubated for 10 min at room temperature (RT), and centrifuged at 600 *g* for 5 min at RT. Pellet was resuspended in PBS with 0.2% BSA. Rh103 nucleic acid intercalator (Fluidigm) was added to cells (0.125 nM) before formaldehyde fixation (Thermo Scientific). Samples were barcoded using Cell-ID 20-plex Pd Barcoding Kit (Fluidigm) and labeled with a cocktail of antibodies to identify major immune subsets (Supplementary Table 4). Acquisition and data analysis was performed as described for PBMCs.

### Immunohistological analysis of atherosclerotic lesions of *Apoe*^*−/−*^ mice

Aortic root serial cryosections (5 µm thick) were cut and stained for macrophages (rat antimouse CD68, dilution 1:25; catalog no. MCA1957; Bio-Rad) or T cells (rabbit antimouse CD3, dilution 1:50; catalog no. MA1-90582; Thermo Scientific). After primary antibody incubation (overnight at 4 °C) either a secondary antirat IgG horseradish peroxidase-conjugated antibody (dilution 1:100; catalog no. STAR72; Bio-Rad) or goat antirabbit IgG horseradish peroxidase-conjugated antibody (dilution 1:100; catalog no. 1705046; Bio-Rad) were incubated for 1 h at RT. Peroxidase activity was detected using 3,3′-diaminobenzidine tablets (D4293; Sigma-Aldrich), and sections were counterstained with hematoxylin (catalog no. 3530-32; Ricca Chemical). Negative control was performed by omitting the primary antibody. Images were acquired using the BZ-X800 microscope (Keyence), and the quantification of stained area was performed with Image Pro Plus (v.9; Media Cybernetics). Results were expressed as average of three to five sections per mouse. Normal distribution of the data was confirmed using the Shapiro–Wilk test and Kolmogorov–Smirnov test. Statistical analysis was performed by one-way ANOVA with Tukey’s post hoc test.

### En face analysis of the aorta

Mouse aortas were cleaned and put in 10% formalin (catalog no. 9990244; Thermo Scientific) for 24–72 h at 4 °C. The aorta was cut open and pinned on 7% agarose (catalog no. 17852; Thermo Scientific) for en face analysis using ORO (stock, 1 g ORO (catalog no. O0625; Sigma-Aldrich) + 200 ml isopropyl alcohol (catalog no. A416-500; Fisher Chemical)). ORO^+^ stained area was quantified using Image Pro Plus (v.9; Media Cybernetics) and data were expressed as the percentage of total aortic area. After Shapiro–Wilk and Kolmogorov–Smirnov tests, statistical analysis was performed using one-way ANOVA with Tukey’s post hoc test.

### RNA extraction from mouse aorta

Mouse atherosclerotic aortas were stored in RNAlater (catalog no. AM7021; Invitrogen) for 24 hours at 4 °C, and tissue was then stored at −80 °C until processing for RNA extraction using the gentleMACS Octo Dissociator (Miltenyi Biotec) homogenization protocol for total RNA isolation, the QIAzol Lysis Reagent (catalog no. 79306; Qiagen) and RNA clean-up using the RNAeasy Mini Kit (Qiagen). Total RNA was isolated with the RNeasy Mini Kit (catalog no. 74104; Qiagen). Sample quality and quantity was determined with the Agilent 2100 bioanalyzer, using the Agilent RNA 6000 Nano Kit (catalog no. 5067-1511; Agilent Technologies). RNA (250 ng) was used as input for poly(A) library construction, and RNA-seq was performed with the DNBseq platform (BGI Americas Corporation).

### RNA-seq analysis of mouse aortas

For analysis on mouse samples, RNA-seq raw counts were log_2_-transformed and *z*-score scaled. Unbiased hierarchical clustering was performed on the top 100 most variable genes across all samples after drop out of outlier and low-expressing genes using Clustergrammer2 (https://github.com/ismms-himc/clustergrammer2). DEGs were submitted to Enrichr. The pathways from Enrichr were ranked based on the combined score, which represents the *P* value (Fisher’s exact test) multiplied by the *z*-score of the deviation from the expected rank. GO annotations and signaling pathways were represented as bar graphs.

### Seahorse experiments

Two 16-week-old male C57BL/6 mice (Jackson Laboratories) were fed a standard diet and housed (12-h light–dark cycle conditions; temperature 20–24 °C, 30–70% humidity) at the NYU Grossmann School of Medicine. BMDMs were differentiated from isolated bone marrow cells as described^66^. BMDMs were plated at 200,000 cells per well and treated with vehicle (DMSO < 0.1% v/v), saracatinib (0.1, 1 or 10 µM) with or without oxLDL (50 µg ml^−1^) in DMEM with 2% FBS for 5 h. Media was replaced 1 h before measurement with unbuffered assay medium supplemented with 2 mM pyruvate, 1 mM glutamine, 10 mM glucose, and with either vehicle (DMSO < 0.1% v/v) or saracatinib (0.1 µM) with or without oxLDL (50 µg ml^−1^). OCR was assessed by Cell Mito Stress Test (Agilent technologies). Statistical analysis was performed by one-way ANOVA with Dunnet’s post hoc test versus untreated control cells (Prism v.9 for MacOS).

### Rabbit model of atherosclerosis

The rabbit study was designed to test the hypothesis that saracatinib could reduce atherosclerosis inflammation progression by [^18^F]FDG PET–MRI. The sample size calculation was based on historical data on the effect of atorvastatin on plaque inflammation measured by [^18^F]FDG PET–MRI in rabbits and an estimated effect size of 0.19, *ɑ* of 0.05 and *σ* of 0.08 to have a power of 80%. The calculated sample size was six animals per group.

Male New Zealand white rabbits (*n* = 40, NZW/LacJ, 2.5 kg; strain no. 001058; Jackson Laboratories) were housed at the Icahn School of Medicine at Mount Sinai (12-h light–dark cycle conditions; temperature 20–24 °C, 30–70% humidity) and fed a WD (4.7% hydrogenated coconut oil and 0.3% cholesterol; catalog no. C30255; Research Diet). An experimental flow diagram is included in Supplementary Fig. 6. Rabbits underwent two aortic endothelial denudations at 2 and 6 weeks after diet initiation, as described in refs. ^46,48^. At 8 weeks, animals were switched to a 0.15% cholesterol-enriched WD (4.7% hydrogenated coconut oil and 0.15% cholesterol; catalog no. C30298Y; Research Diet). At the end of the 16-week atherosclerosis induction period, rabbits underwent baseline [^18^F]FDG PET–MRI and were randomized in four treatment groups of treatment for 12 weeks: (1) 0.15% cholesterol-enriched WD, (2) 0.15% cholesterol-enriched WD plus 3 mg kg^−1^ d^−1^ atorvastatin (catalog no. C17120401; Research Diet), (3) 0.15% cholesterol-enriched WD plus 4 mg kg^−1^ d^−1^ saracatinib (catalog no. C18082901; Research Diet) and (4) 0.15% cholesterol-enriched WD plus saracatinib and atorvastatin (catalog no. C18082902; Research Diet). The drugs used were the same stocks used for the mouse diet preparation. Venous blood was collected at baseline and at 16 and 28 weeks in lithium heparin. Rabbits were killed at week 28 using sodium phenobarbital (100 mg kg^−1;^ catalog no. 078059296; Patterson Veterinary Supply). Rabbit aortas were perfused with 1,000 ml of 0.9% sodium chloride solution (catalog no. 2B1324X; Baxter) and harvested. Plasma was isolated by centrifugation at 1,699 *g* at 4 °C and stored at −80 °C to measure circulating total cholesterol levels using the Cholesterol E Kit (catalog no. 999-02601; FUJIFILM Wako Diagnostics). Complete blood count from rabbits at 28 weeks was performed using the Coulter Ac-T 5diff Hematology Analyzer (Beckman Coulter). Normal distribution of the data was confirmed using the Shapiro–Wilk test and Kolmogorov–Smirnov test, and statistical analysis was performed using one-way ANOVA with Tukey’s post hoc test (Prism v.9 for MacOS).

### In vivo [^18^F]FDG PET–MRI in rabbits

In vivo imaging of rabbits was performed on a 3 T Biograph mMR (Siemens) PET–MRI clinical scanner using a product six-channel body array^67^. Rabbits were fasted for 3–4 h before injection of [^18^F]FDG (5 mCi) in the marginal ear vein^48^. Rabbits were then anesthetized intramuscularly with ketamine (20 mg kg^−1^) and xylazine (5 mg kg^−1^), and the bladder was emptied with a catheter. Animals were then placed in a supine position under 1.5% isoflurane inhalation on the PET–MRI scanner and vitals were monitored. After scout scans, PET imaging was initiated for 30 min. [^18^F]FDG PET images were reconstructed offline using a three-dimensional ordinary Poisson ordered subset expectation maximization algorithm with point-spread-function resolution modeling, using 3 iterations and 21 subsets and filtered with a 4-mm Gaussian filter. Three-dimensional noncontrast-enhanced time-of-flight (TOF) magnetic resonance images were acquired to visualize the abdominal aorta and renal arteries, with the following imaging parameters: repetition time, 23 ms; echo time, 2.8 ms; flip angle, 20°; spatial resolution, 0.35 × 0.35 mm^2^ (interpolated); and slice thickness, 1 mm. As TOF images were acquired in three-dimensional mode, there was no gap in between slices. A three-dimensional T2-weighted Sampling Perfection with Application optimized Contrasts using different flip angle Evolution (SPACE) magnetic resonance sequence was acquired to quantify vessel wall area. Images were acquired in a sagittal slab, with slice selective excitation, and readout in the foot to head direction, to allow for extensive coverage from above the renal arteries, down to the iliac bifurcation. Imaging parameters were repetition time, 1,600 ms; echo time, 113 ms; excitation flip angle, 90°; variable refocusing flip angle(s); acquired spatial resolution, 0.63 × 0.63 × 0.63 mm^3^; and no interpolation was used.

PET images were fused with TOF bright-blood MRI angiography and regions of interest were manually drawn on abdominal aorta from the left renal artery to the iliac bifurcation in TOF images using OsiriX v.5.6 software (OsiriX Foundation). Standard windowing of the PET signal was used, based on evaluation of the skeletal muscle. The slice-by-slice SUVmax values were averaged across the whole aorta^48^. To ensure blinding, imaging analysts were not aware of the group distribution. For MRI, slice-by-slice inner and outer vessel wall were traced on axially reformatted three-dimensional T2-weighted SPACE images. Vessel wall area (cm^2^) was calculated as the difference between outer and inner wall area, and averaged from below the left renal artery down to the iliac bifurcation^46^.

### Histological analysis of the atherosclerotic rabbit aorta

For histological analysis, the imaged abdominal aorta was dissected, sectioned in eight pieces and put into 10% formalin for 24 h at 4 °C, then in 20% sucrose for the next 24 h at 4 °C, before being embedded in OCT and stored at −80 °C. Five 5-µm-thick sections were acetone-fixed sections (catalog no. HC-300; Fisher Scientific), incubated with 3% hydrogen peroxide (catalog no. H325-100; Fisher Scientific) at RT for 20 min and blocked with 3% BSA (catalog no. 001-000-162; Jackson ImmunoResearch) plus 3% goat serum (catalog no. X0907; Agilent Dako). Sections were then incubated with an anti-RAM11 mouse monoclonal antibody (dilution 1:50; catalog no. M0633; Agilent Dako) for 2 h at 37 °C, and with an antimouse secondary antibody (catalog no. HK335-9M; BioGenex) for 15 min at 37 °C. Streptavidine peroxidase (catalog no. HK330-9K; BioGenex) was incubated for 10 min at 37 °C, and peroxidase activity was detected using 3,3′-diaminobenzidine (catalog no. D3939; Sigma-Aldrich). Sections were counterstained with hematoxylin (catalog no. 3530-32; Ricca Chemical). ORO staining was performed as described for the mouse aortas. Images were acquired using the BZ-X800 microscope (Keyence), and quantification of positively stained areas was performed with the BZ-X800 Analyzer and BZ-H4C Hybrid Cell Count Software (Keyence). Macrophages were quantified as the percentage of total vessel wall area positive for RAM11 staining. ORO^+^ areas were quantified as the percentage of total vessel wall. Results were expressed as an average of 3–5 sections per animal. Normal distribution of the data was confirmed using the Shapiro–Wilk test and Kolmogorov–Smirnov test. Statistical analysis was performed by two-sample paired *t*-test and one-way ANOVA with Tukey’s post hoc across all groups (Prism v.9 for MacOS).

### Reporting summary

Further information on research design is available in the Nature Portfolio Reporting Summary linked to this article.

### Supplementary information

Supplementary Information Supplementary Figs. 1–6.

Reporting Summary

Supplementary Tables Supplementary Tables 1–4.

## Data Availability

RNA-seq raw data are available in the Gene Expression Omnibus (GEO:GSE230217). CyTOF raw data are available at https://zenodo.org/record/7851084#.ZELm5OzMJw9. All other data supporting the findings in this study are available at https://github.com/giannarelli-lab/Systems-immunology-based-drug-repurposing-framework-to-target-inflammation-in-atherosclerosis.

## Code availability

Code used for data analysis in this manuscript is available on GitHub at https://github.com/giannarelli-lab/Systems-immunology-based-drug-repurposing-framework-to-target-inflammation-in-atherosclerosis.
