# Publisher Correction: Systems immunology-based drug repurposing framework to target inflammation in atherosclerosis

**DOI:** 10.1038/s44161-023-00310-1

**Published:** 2023-07-04

**Authors:** Letizia Amadori, Claudia Calcagno, Dawn M. Fernandez, Simon Koplev, Nicolas Fernandez, Ravneet Kaur, Pauline Mury, Nayaab S Khan, Swathy Sajja, Roza Shamailova, Yannick Cyr, Minji Jeon, Christopher A. Hill, Peik Sean Chong, Sonum Naidu, Ken Sakurai, Adam Ali Ghotbi, Raphael Soler, Natalia Eberhardt, Adeeb Rahman, Peter Faries, Kathryn J. Moore, Zahi A. Fayad, Avi Ma’ayan, Chiara Giannarelli

**Affiliations:** 1 Department of Medicine, Division of Cardiology, NYU Cardiovascular Research Center, New York, NY USA; 2 The Icahn Institute for Genomics and Multiscale Biology, Icahn School of Medicine at Mount Sinai, New York, NY USA; 3 BioMedical Engineering and Imaging Institute, Icahn School of Medicine at Mount Sinai, New York, NY USA; 4 Department of Diagnostic, Molecular and Interventional Radiology, Icahn School of Medicine at Mount Sinai, New York, NY USA; 5 Department of Medicine, Division of Cardiology, Icahn School of Medicine at Mount Sinai, New York, NY USA; 6 Mount Sinai Center for Bioinformatics, Department of Pharmacological Sciences, Icahn School of Medicine at Mount Sinai, New York, NY USA; 7 Precision Immunology Institute, Icahn School of Medicine at Mount Sinai, New York, NY USA; 8 Department of Surgery, Vascular Division, Icahn School of Medicine at Mount Sinai, New York, NY USA; 9 Department of Pathology; NYU Grossman School of Medicine, NYU Langone Health, New York, NY USA

**Keywords:** Systems analysis, Cardiology, Immunotherapy, Drug discovery, Dyslipidaemias

Correction to: *Nature Cardiovascular Research* 10.1038/s44161-023-00278-y. Published online 8 June 2023.

In the version of this article initially published, a protein (pMAPKAPK2) was misspelled in Fig. 1 and Extended Data Fig. 7; a colored box for “*AKT*” was missing from the second column of regulators in Fig. 5a; Extended Data Fig. 2f was missing a header above the color key; and typographical errors (extraneous citations to refs. 1, 3 and 5) were present in the “Analysis of RNA-seq data from saracatinib-treated tissue” section of Methods. In addition, the Reporting Summary and the legends for Supplementary Figs. 5 and 8 were outdated versions. The errors have been corrected in the HTML and PDF versions of the article.

