# Supplementary material for: Systems immunology-based drug repurposing framework to target inflammation in atherosclerosis
Source: Nat Cardiovasc Res. 2023 Jun 8;2(6):550–71. doi: 10.1038/s44161-023-00278-y (PMC10538622; doi:10.1038/s44161-023-00278-y)
Supplement: Supplementary file 1 — Supplementary Figs. 1–6. [file 44161_2023_278_MOESM1_ESM.pdf]

nature cardiovascular research

Article https://doi.org/10.1038/s44161-023-00278-y

Systems immunology-based drug repurposing framework to target inflammation in atherosclerosis

In the format provided by the authors and unedited

Supplementary Data Figure 1. Phospho-CyTOF analysis of intracellular signaling in healthy PBMCs stimulated with either atherosclerotic or healthy plasma. Dot plots show the effect of atherosclerotic plasma (athero, n=20 biologically independent samples; males=10), healthy plasma (healthy, n=10 biologically independent samples) or no stimulation (no plasma, n=5 biologically independent samples) on the phosphorylation of intracellular kinases in **A.** $CD14^+$ monocytes, **B.** $CD1c^+$ DCs, **C.** $CD16^+$ monocytes, **D.** CD4+ T cells and **E.** CD8+ T cells from healthy PBMCs. P values were determined by one-way ANOVA with Tukey's post hoc test across all groups.

Supplementary Data Figure 1

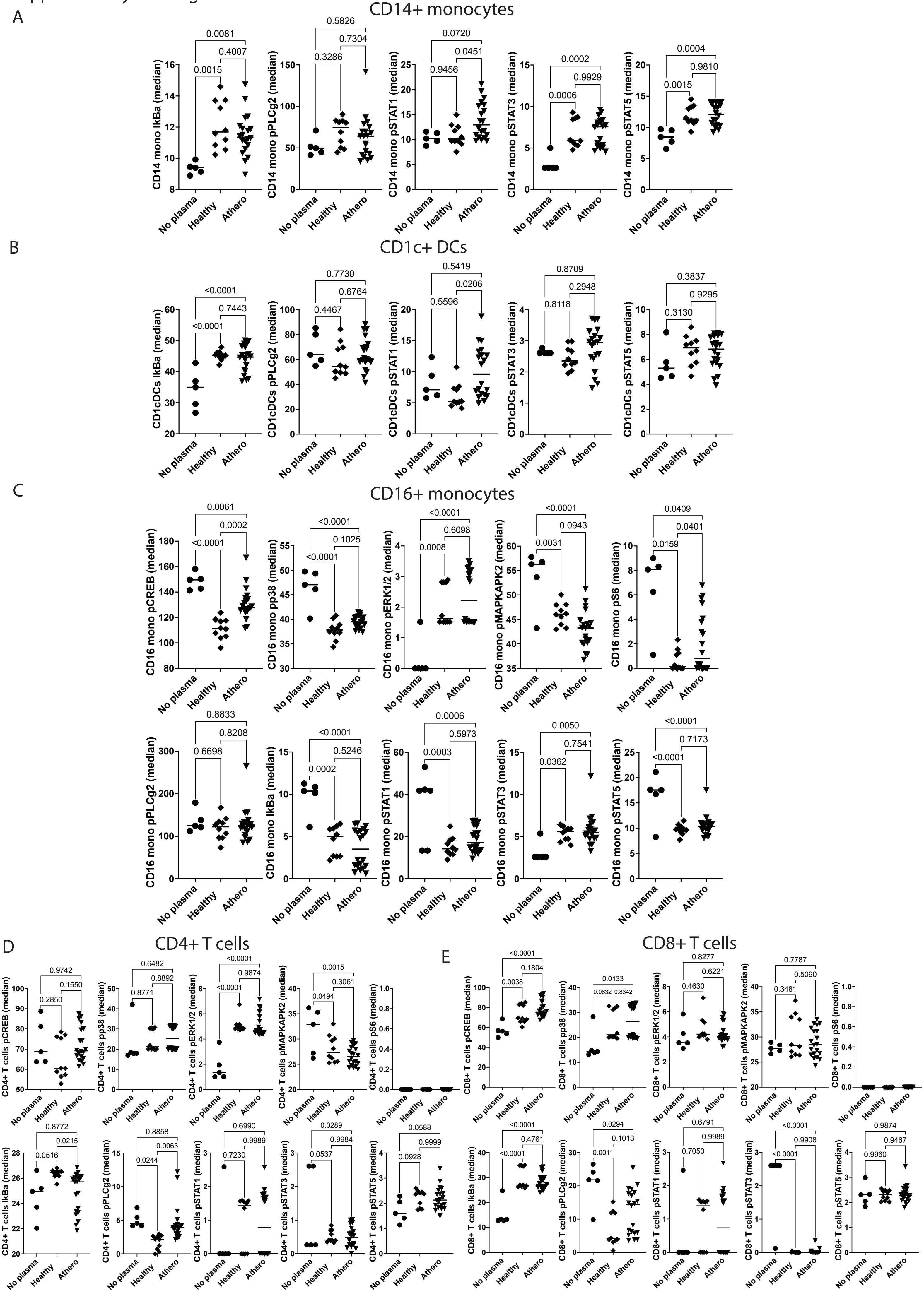

Supplementary Data Figure 2. Phospho-CyTOF analysis of intracellular signaling in PBMCs from atherosclerotic patients stimulated with either autologous or healthy plasma. Dot plots show the effect of autologous atheroplasma (auto, n=9 biologically independent samples; males=5) vs. healthy plasma (healthy, n=9 biologically independent samples) or no stimulation (no plasma, n=10 biologically independent samples; males=6) on the phosphorylation of intracellular kinases in **A.** $CD14^+$ monocytes, **B.** $CD1c^+$ DCs, **C.** $CD16^+$ monocytes, **D.** CD4+ T cells and **E.** CD8+ T cells in PBMCs from atherosclerotic patients. P values were determined by one-way ANOVA with Tukey's post hoc test across all groups.

Supplementary Data Figure 2

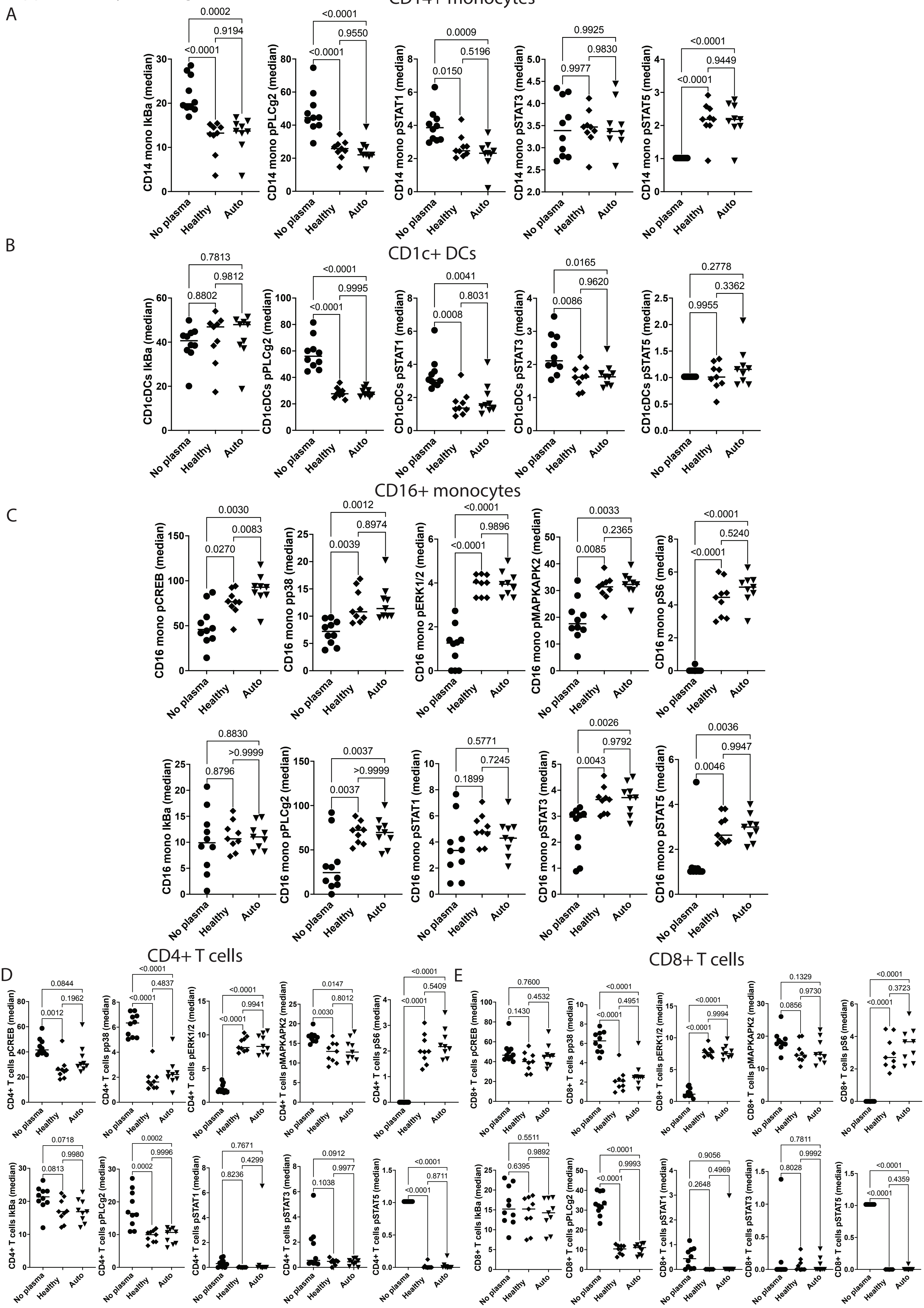

Supplementary Data Figure 3. Phospho-CyTOF analysis of intracellular signaling in CD14+ monocytes, CD1c+ DCs and CD16+ monocytes subpopulations of healthy PBMCs stimulated with oxLDL. Dot plots show the effect of oxLDL (50µg/ml), atherosclerotic plasma (athero), atherosclerotic plasma + oxLDL (athero+oxLDL), or no stimulation on the phosphorylation of intracellular kinases in **A**. $CD14^+$ monocytes, **B.** CD1c+ DCs and **C.** $CD16^+$ monocytes in PBMCs from healthy donors. N=4 biologically independent samples/condition. P values were determined by one-way ANOVA with Tukey's post hoc test across all groups.

Supplementary Data Figure 3

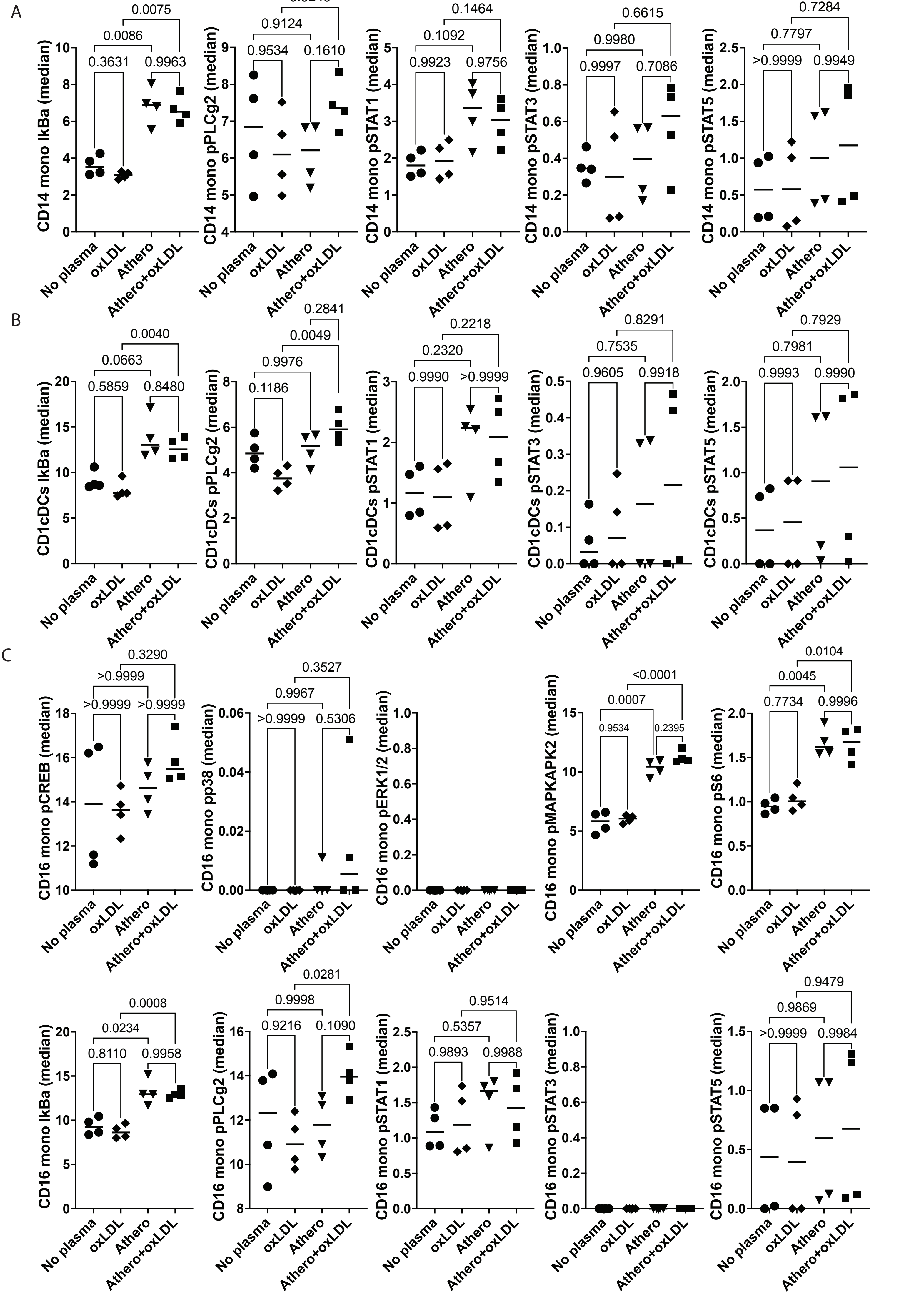

Supplementary Data Figure 4. Phospho-CyTOF analysis of intracellular signaling in CD4+ T cells and CD8+ T cells subpopulations of healthy PBMCs stimulated with atherosclerotic plasma in presence of oxLDL. Dot plots show the effect of oxLDL (50µg/ml), atherosclerotic plasma (athero), atherosclerotic plasma + oxLDL (athero+oxLDL), or no stimulation on the phosphorylation of intracellular kinases in **A**. CD4+ T cells and **B.** CD8+ T cells. N=4 biologically independent samples/condition. P values were determined by one-way ANOVA with Tukey's post hoc test across all groups.

Supplementary Data Figure 4

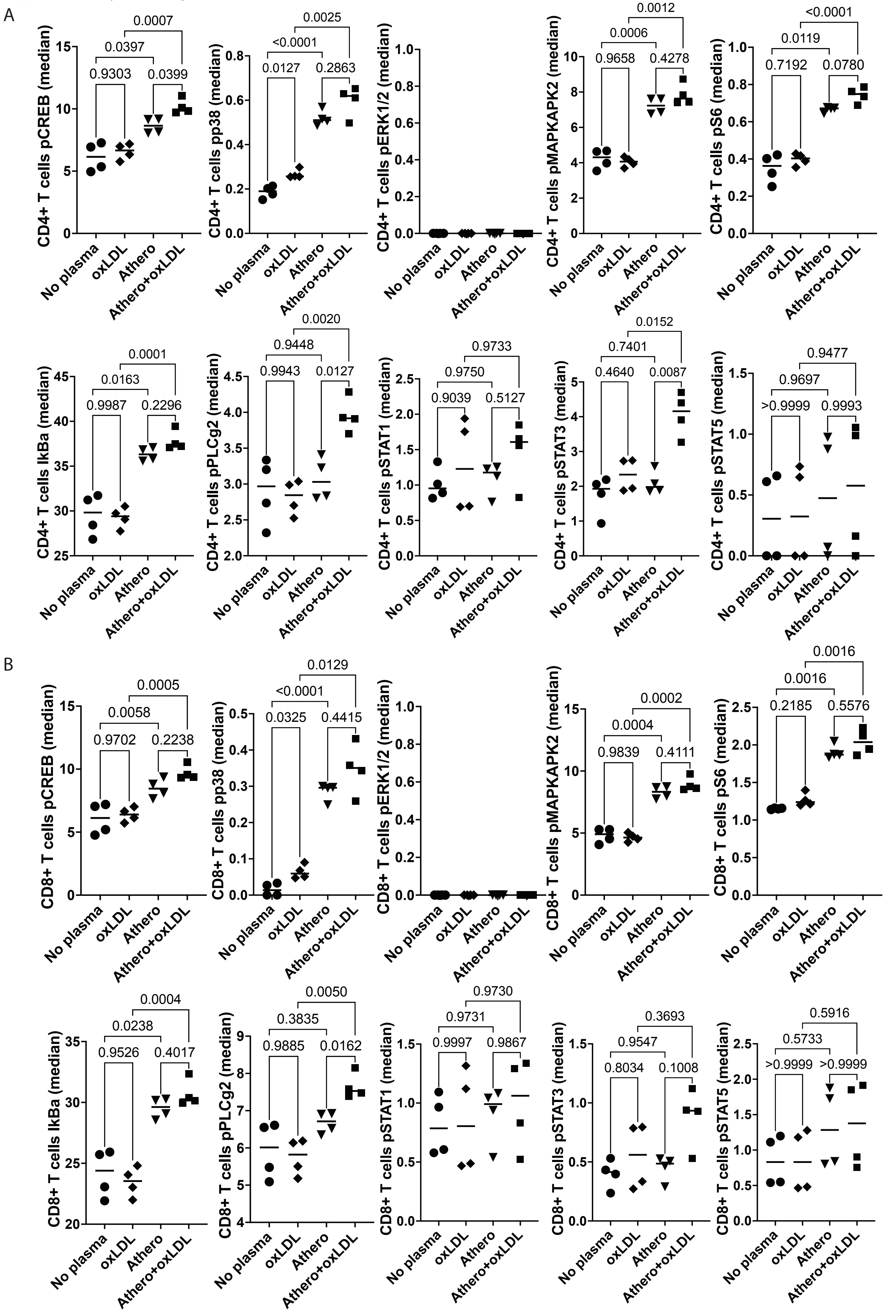

Supplementary Data Figure 5. Effects of saracatinib on intracellular signaling and metabolic pathways induced by autologous atheroplasma in PBMCs from patients and in bone marrow derived mouse macrophages. A. Half maximal inhibitory concentration values (IC50) of saracatinib (expressed as log2 values) used to treat $CD14^+$ monocytes exposed to plasma from atherosclerotic patients and analyzed by CyTOF to measure the inhibition of each phosphosite induced by saracatinib (% signal reduction). **B.** Effect of saracatinib (25 μM) on $CD14^+$ monocytes and DCs from patients with coronary artery disease (CAD, n=5 biologically independent samples; males=3) or carotid artery disease (CEA, n=5 biologically independent samples; males=3) measured as the % of change in phospho-signaling by CyTOF. P-values were determined using Welch's unpaired t-test, two-tailed. **C.** Oxygen consumption rate (OCR) and respiratory parameters in bone marrow derived mouse macrophages treated with vehicle (DMSO) or saracatinib (sara) 0.1-1-10μM and summary of respiratory parameters: Basal OCR, Adenosine 5′-triphosphate (ATP) production, Spare Respiratory capacity (Spare Resp Cap): DMSO, n=5; saracatinib 0.1μM, n=5; saracatinib 1μM, n=5; saracatinib 10μM, n=4 biologically independent samples. Maximal Respiratory OCR: DMSO, n=5; saracatinib 0.1μM, n=5; saracatinib 1μM, n=5; saracatinib 10μM, n=5 biologically independent samples. P values were determined by one-way ANOVA with Dunnet's post hoc test vs vehicle. Data are presented as mean values +/- SD.

D. Oxygen consumption rate (OCR) and respiratory parameters in bone marrow derived mouse macrophages treated with vehicle plus oxLDL (DMSO+ oxLDL) or saracatinib (sara, 0.1-1-10μM) + oxLDL and summary of respiratory parameters in bone marrow derived mouse macrophages: Basal OCR: DMSO+oxLDL, n=4; saracatinib 0.1μM+oxLDL, n=5; saracatinib 1μM+oxLDL, n=5; saracatinib 10μM+oxLDL, n=5 biologically independent samples. Adenosine 5′-triphosphate (ATP) production, Maximal Respiratory OCR, Spare Respiratory capacity (Spare Resp Cap): DMSO+oxLDL, n=5; saracatinib 0.1μM+oxLDL, n=5; saracatinib 1μM+oxLDL, n=5; saracatinib 10μM+oxLDL, n=5 biologically independent samples. P values were determined by one-way ANOVA with Dunnet's post hoc test vs vehicle. Data are presented as mean values +/- SD.

Supplementary Data Figure 5

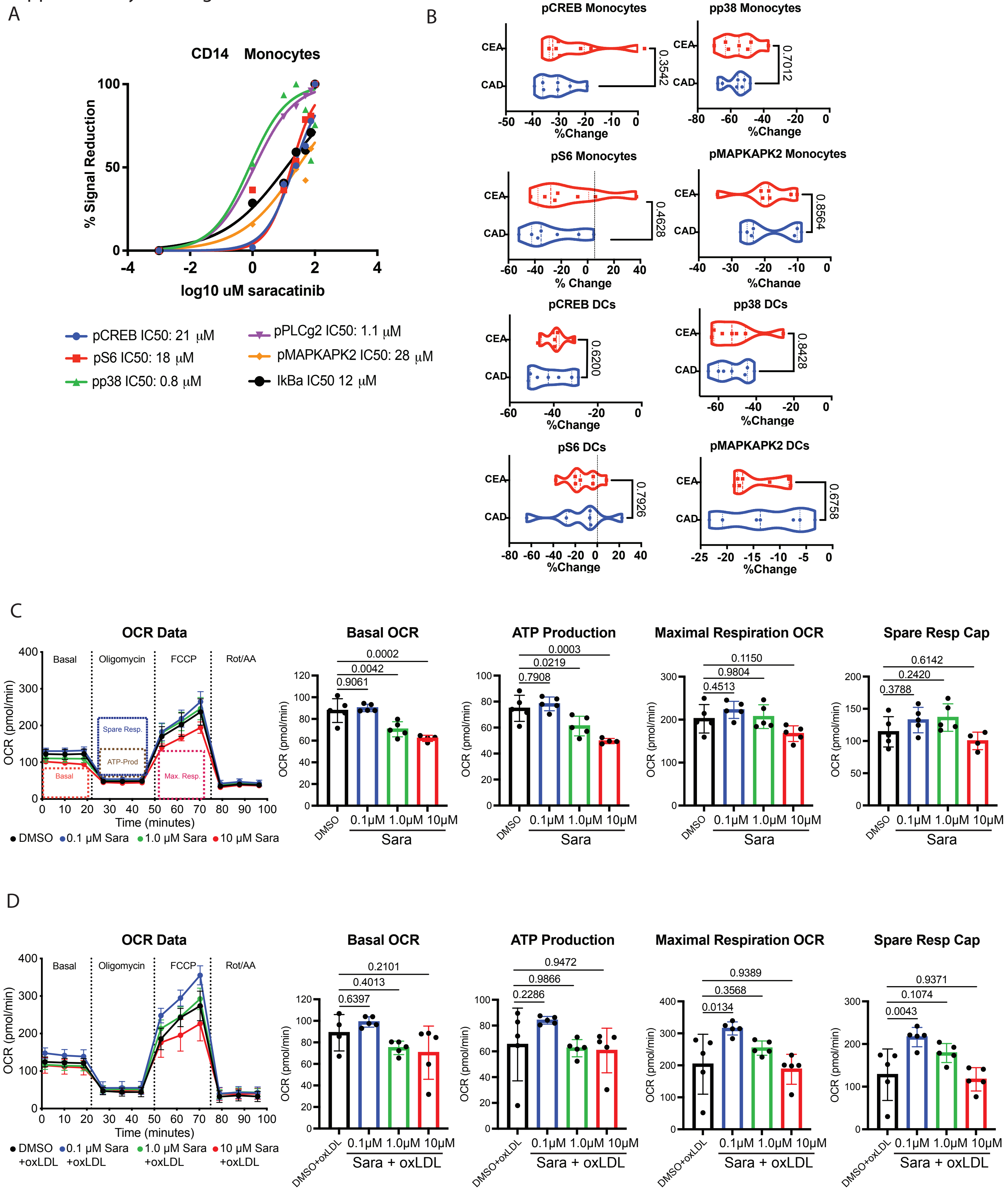

Supplementary Data Figure 6. Flow diagram of rabbit study. The flow diagram depicts the experimental design of the rabbit study and shows animals that were included and excluded as part of the study.

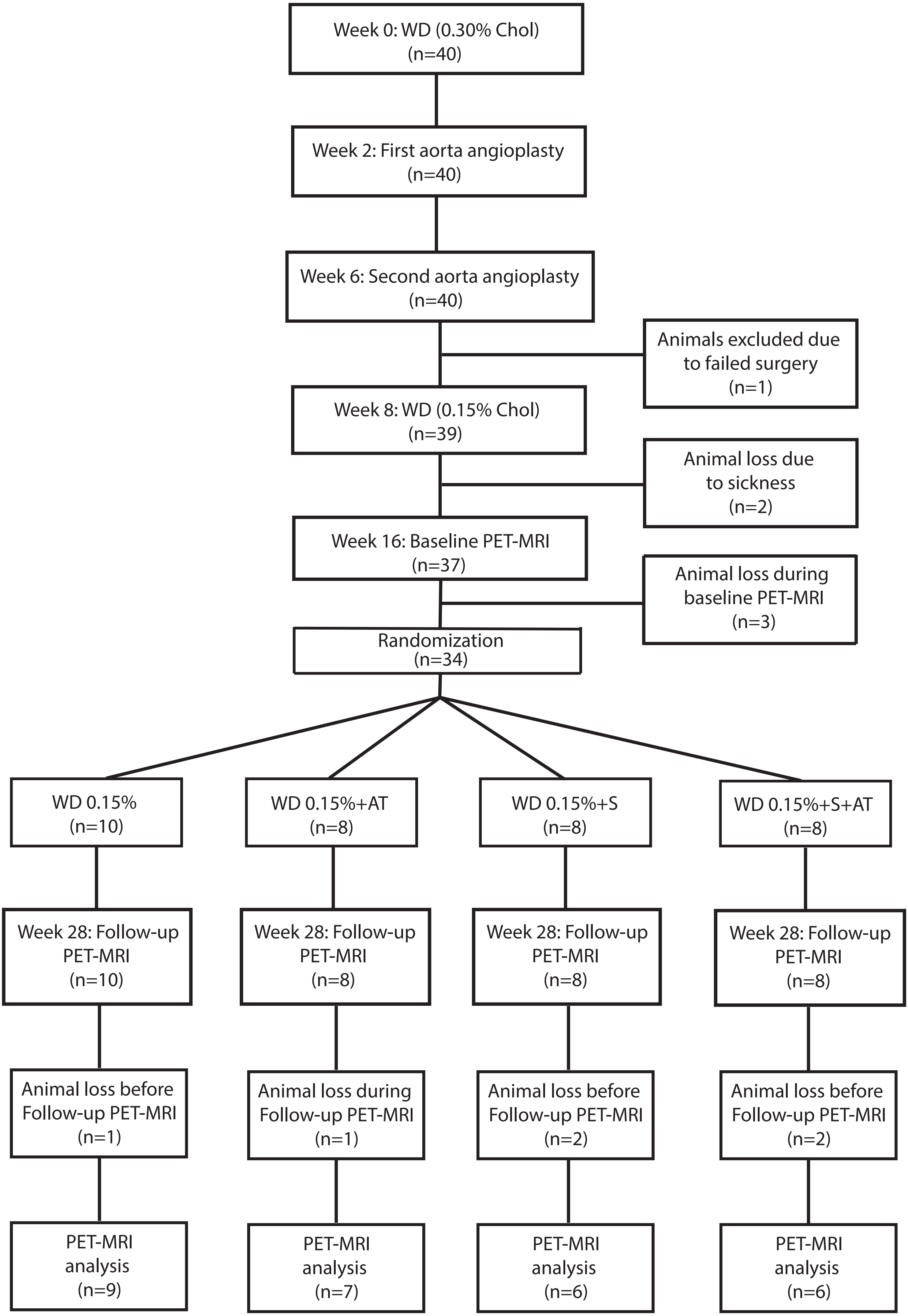
Week 0: WD (0.30% Chol)
(n=40)
Week 2: First aorta angioplasty
(n=40)
Week 6: Second aorta angioplasty
(n=40)
Animals excluded due to failed surgery
(n=1)
Week 8: WD (0.15% Chol)
(n=39)
Animal loss due to sickness
(n=2)
Week 16: Baseline PET-MRI
(n=37)
Animal loss during baseline PET-MRI
(n=3)
Randomization
(n=34)
WD 0.15%
(n=10)
WD 0.15%+AT
(n=8)
WD 0.15%+S
(n=8)
WD 0.15%+S+AT
(n=8)
Week 28: Follow-up PET-MRI
(n=10)
Week 28: Follow-up PET-MRI
(n=8)
Week 28: Follow-up PET-MRI
(n=8)
Week 28: Follow-up PET-MRI
(n=8)
Animal loss before Follow-up PET-MRI
(n=1)
Animal loss during Follow-up PET-MRI
(n=1)
Animal loss before Follow-up PET-MRI
(n=2)
Animal loss before Follow-up PET-MRI
(n=2)
PET-MRI analysis
(n=9)
PET-MRI analysis
(n=7)
PET-MRI analysis
(n=6)
PET-MRI analysis
(n=6)
