# Supplementary material for: Systems immunology-based drug repurposing framework to target inflammation in atherosclerosis
Source: Nat Cardiovasc Res. 2023 Jun 8;2(6):550–71. doi: 10.1038/s44161-023-00278-y (PMC10538622; doi:10.1038/s44161-023-00278-y)
Supplement: Supplementary file 2 — Reporting Summary [file 44161_2023_278_MOESM2_ESM.pdf]

nature portfolio

Corresponding author(s): Chiara Giannarelli

Last updated by author(s): May 23, 2023

Reporting Summary

Nature Portfolio wishes to improve the reproducibility of the work that we publish. This form provides structure for consistency and transparency in reporting. For further information on Nature Portfolio policies, see our Editorial Policies and the Editorial Policy Checklist.

Statistics

For all statistical analyses, confirm that the following items are present in the figure legend, table legend, main text, or Methods section.

n/a	Confirmed	
☐	☒	The exact sample size (*n*) for each experimental group/condition, given as a discrete number and unit of measurement
☐	☒	A statement on whether measurements were taken from distinct samples or whether the same sample was measured repeatedly
☐	☒	The statistical test(s) used AND whether they are one- or two-sided<br>*Only common tests should be described solely by name; describe more complex techniques in the Methods section.*
☐	☒	A description of all covariates tested
☐	☒	A description of any assumptions or corrections, such as tests of normality and adjustment for multiple comparisons
☐	☒	A full description of the statistical parameters including central tendency (e.g. means) or other basic estimates (e.g. regression coefficient) AND variation (e.g. standard deviation) or associated estimates of uncertainty (e.g. confidence intervals)
☐	☒	For null hypothesis testing, the test statistic (e.g. F, t, r) with confidence intervals, effect sizes, degrees of freedom and P value noted<br>*Give P values as exact values whenever suitable.*
☒	☐	For Bayesian analysis, information on the choice of priors and Markov chain Monte Carlo settings
☐	☒	For hierarchical and complex designs, identification of the appropriate level for tests and full reporting of outcomes
☒	☐	Estimates of effect sizes (e.g. Cohen's *d*, Pearson's *r*), indicating how they were calculated

Our web collection on statistics for biologists contains articles on many of the points above.

Software and code

Policy information about availability of computer code

Data collection	CyTOF raw data was collected using the Helios normalizer software (Fluidigm version 6.7.1014). RNA sequencing<br>data was collected using custom code, generated in R v.4.0.3, was used to analyze the rest of the raw data.<br>Code used for data analysis in this manuscript is available on Github under https://github.com/giannarelli-lab/Systems-immunology-based-drug-repurposing-framework-to-target-inflammation-in-atherosclerosis.
Data analysis	CyTOF data was analyzed using Cytobank (https://mtsinai.cytobank.org/ cytobank/; Cytobank, Menlo Park, CA, 7.0) and using custom scripts in R v.4.0.3.<br>Luminex: Milliplex Analyst 5.1 software (EMD Millipore, Billerica, Massachusetts) or Xponent software (Luminex Corporation); R (v.4.0.3).<br>Heatmap in Figure 7 was created using https://clustergrammer.readthedocs.io/<br>RNASeq: Fastqc v0.11.7, STAR v2.6.1d, Subread v1.6.3, DESeq2 R package (1.30.1), Pheatmap package (v1.0.12), Pathway analysis was done using Enrichr v3. (https://amp.pharm.mssm.edu/Enrichr3)<br>Graphpad prism 9 was also used for statistical analysis as described in the Methods.

For manuscripts utilizing custom algorithms or software that are central to the research but not yet described in published literature, software must be made available to editors and reviewers. We strongly encourage code deposition in a community repository (e.g. GitHub). See the Nature Portfolio guidelines for submitting code & software for further information.

Data

Policy information about availability of data

All manuscripts must include a data availability statement. This statement should provide the following information, where applicable:

- Accession codes, unique identifiers, or web links for publicly available datasets
- A description of any restrictions on data availability
- For clinical datasets or third party data, please ensure that the statement adheres to our policy

Human Genome (GRCh38, Gencode v24); RNAseq raw data is available in Gene Expression Omnibus (GEO) under accession number GSE230217. CyTOF raw data is available in https://zenodo.org/record/7851084#.ZELm5OzMJw9. All other data supporting the findings in this study is available in https://github.com/giannarelli-lab/Systems-immunology-based-drug-repurposing-framework-to-target-inflammation-in-atherosclerosis.

Human research participants

Policy information about studies involving human research participants and Sex and Gender in Research.

Reporting on sex and gender	We did not actively select the sex or gender of prospectively enrolled patients. The sex distribution reflects the prevalence of the disease in males.
Population characteristics	Our human study does not qualify as a clinical trial according to the NIH clinical trial definition. We utilized plasma and PBMCs from patients with atherosclerosis and healthy subjects. The clinical characteristics of the patients are reported in Supplementary table 1.
Recruitment	Patients undergoing carotid endarterectomy surgery were prospectively recruited for this study and required to give informed written consent. Exclusion criteria were: current infection, autoimmune disease, active or recurrent cancer, severe renal failure requiring dialysis, or presented with peripheral arterial disease. Patients were prospectively enrolled from patients undergoing surgical revascularization or cardiac angiogram at our Institution at any given day to exclude any potential bias, and exclusively based on the inclusion and exclusion criteria stated in the consent form.
Ethics oversight	PPHS office of the Icahn School of Medicine at Mount Sinai. The human research study approved by the Institutional Review Board (IRB) of the Icahn School of Medicine at Mount Sinai (IRB#11-01427) and the IRB of the NYU Langone Health and the NYU Grossman School of Medicine (IRB#i21-00429)

Note that full information on the approval of the study protocol must also be provided in the manuscript.

Field-specific reporting

Please select the one below that is the best fit for your research. If you are not sure, read the appropriate sections before making your selection.

☒ Life sciences ☐ Behavioural & social sciences ☐ Ecological, evolutionary & environmental sciences

For a reference copy of the document with all sections, see nature.com/documents/nr-reporting-summary-flat.pdf

Life sciences study design

All studies must disclose on these points even when the disclosure is negative.

Sample size	For the human studies the sample size was based on an interim analysis on pilot data.<br>For the mouse studies: No statistical methods were used to pre-determine sample size but our sample sizes are similar to those reported in previous publications (doi:10.1038/s41586-019-0948-2, doi:10.1038/s41419-020-2229-2)<br>For the Rabbit Studies: The sample size calculation was based on historical data on the effect of atorvastatin on plaque inflammation measured by 18F-FDG-PET MRI in rabbits and an estimated effect size of 0.19, alpha: 0.05, sigma: 0.08 to have a power of 80%. The calculated sample size was 6 animals/group.
Data exclusions	We did not exclude any data from the dataset.
Replication	For IHC, en face and Oil Red O quantification, all samples were quantified for a minimum of three sections/whole aortas and quantification was performed by two blinded independent observers. For PET-MRI analysis, a minimum of three animals/group were used for quantification that was performed by two blinded independent investigators. For CyTOF, Luminex, RNA-seq analysis, a minimum of two or three samples/group or condition were used. All attempts at replication were successful.
Randomization	For human studies we did not actively randomize our patients, as the specimens were processed from prospectively enrolled patients. Co-variates analysis was not applicable as demographic/disease characteristics was similar for each comparison.<br>For mouse work, mice were randomized into seven groups before undergoing treatment with Western diet -/+ drugs.<br>For rabbit work, rabbits were randomized into four groups after atherosclerosis induction and before being fed Western diet -/+ drugs.

Blinding

In mouse and rabbit work, the quantification of CD3+, CD68+ cells, RAM11+ cells, OilRedO+ areas and en face analysis were performed blindly. For PET-MRI analysis, to ensure blinding, the investigators involved in the imaging analysis were not aware of the group distribution in the imaging slices. For the studies using human specimens, we used unbiased computational approaches to analyze the data. Researchers processing all data were blinded to the study.

Reporting for specific materials, systems and methods

We require information from authors about some types of materials, experimental systems and methods used in many studies. Here, indicate whether each material, system or method listed is relevant to your study. If you are not sure if a list item applies to your research, read the appropriate section before selecting a response.

Materials & experimental systems

n/a	Involved in the study	
☐	☒	Antibodies
☒	☐	Eukaryotic cell lines
☒	☐	Palaeontology and archaeology
☐	☒	Animals and other organisms
☒	☐	Clinical data
☒	☐	Dual use research of concern

Methods

n/a	Involved in the study	
☒	☐	ChIP-seq
☒	☐	Flow cytometry
☒	☐	MRI-based neuroimaging

Antibodies

Antibodies used

Immunohistochemistry
1. Rat anti mouse CD68 antibody, cat# MCA1957, clone FA-11, lot# 1708, Bio-rad
2. Monoclonal mouse anti-rabbit macrophage clone RAM11, cat# M0633, lot# 11139884, Agilent Dako
3. Goat anti rat IgG:HRP antibody, cat# STAR72, Bio-rad
4. Rabbit anti-mouse CD3, Cat# MA1-90582, Lot# XI3707612, Thermo Scientific
5. goat anti-rabbit IgG HRP-conjugated antibody, Cat# 1705046, Bio-rad
6. anti-mouse HRP-labeled, cat#HK330-9K, BioGenex

7. CyTOF antibodies: a full list is provided in Supplementary Table 3 (human CyTOF panel) and Supplementary Table 4 (mouse CyTOF panel). Antibodies were used at a concentration of 0.2-0.5ug/ ml and were internally controlled by comparison to previous batches of antibodies used at the IMC at Mount Sinai.

7-I. Human CyTOF panel
Elemental isotope Antigen Target (Human) Clone Number Cat# Supplier
113In CD57 HCD57 322325 Biolegend
115In CD45 HI130 304405 Biolegend
116Cd CD45 HI30 3116001B Fluidigm
141Pr CD3 UCHT1 3141019B Fluidigm
142Nd CD19 HIB19 302247 Biolegend
142Nd CD19 HIB19 3142001B Fluidigm
143Nd CD45RA HI100 304143 Biolegend
143Nd CD45RA HI100 3143006B Fluidigm
144Nd CD1c, FITC-conjugated L161 331518 Biolegend
144Nd CD161, FITC-conjugated HP-3G10 339906 Biolegend
144Nd Anti-FITC FIT-22 3144006B Fluidigm
145Nd CD4 RPA-T4 300541 Biolegend
145Nd CD4 RPA-T4 3145001B Fluidigm
146Nd CD8a RPA-T8 301053 Biolegend
146Nd CD8a RPA-T8 3146001B Fluidigm
147Sm p-STAT5 47 3147012A Fluidigm
148Nd CD16 3G8 302051 Biolegend
148Nd CD16 3G8 3148004B Fluidigm
149Sm CD127 A019D5 351337 Biolegend
149Sm CD127 A019D5 3149011B Fluidigm
150Nd CD1c L161 331502 Biolegend
150Nd SRC, Biotin-conjugated SRC-112-BIOTIN Thermo Fisher
150Nd CD1c, Biotin-conjugated L161 331504 Biolegend
150Nd Anti-Biotin 1D4-C5 3150008B Fluidigm
151Eu CD123 6H6 306027 Biolegend
151Eu CD123 6H6 3151001B Fluidigm
152Sm CD66b G10F5 305102 Biolegend
152Sm CD66b G10F5 3152011B Fluidigm
152Sm p-AKT (S473) D9E 3152005A Fluidigm
153Eu p-STAT1 58D6 3153003A Fluidigm
153Eu LCK LCK-01 3153017B Fluidigm
155Gd CD27 O323 302839 Biolegend

155Gd CD27 L128 3155001B Fluidigm
156Gd p-p38 D3F9 3156002A Fluidigm
156Gd AKT, PE-conjugated C67E7 8790S Cell Signaling Technology
156Gd Anti-PE PE001 3156005B Fluidigm
158Gd p-STAT3 4 3158005A Fluidigm
159Tb p-MAPKAPK2 27B7 3159010A Fluidigm
160Gd CD14 M5E2 301843 Biolegend
160Gd CD14 M5E2 3160001B Fluidigm
161Dy CD56 B159 555514 BD Biosciences
162Dy p-PLCg2 K86-689.37 3162018A Fluidigm
162Dy p-LCK 4/LCK-Y505 3162004A Fluidigm
163Dy CD56 (NCAM) NCAM16.2 3163007B Fluidigm
164Dy IkBa L35A5 3164004A Fluidigm
165Ho p-CREB 87G3 3165009A Fluidigm
166Er CD25 M-A251 356102 Biolegend
167Er p-ERK1/2 D13.14.4E 3167005A Fluidigm
168Er CD3 UCHT1 300443 Biolegend
169Tm CD25 (IL-2R) 2A3 3169003B Fluidigm
170Er CD38 HB-7 356602 Biolegend
171Yb CD161 HP-3G10 339919 Biolegend
172Yb CD38 HIT2 3172007B Fluidigm
174Yb HLADR L243 307651 Biolegend
174Yb HLADR L243 3174001B Fluidigm
175Lu p-S6 N7-548 3175009A Fluidigm
176Yb p-SRC, APC- conjugated SrcY416-C4 MA5-36960 Thermo Fisher
176Yb Anti-APC APC003 3176007B Fluidigm
176Yb CD57 HCD57 3176019B Fluidigm

7-II. Mouse CyTOF panel
Elemental Isotope Antigen Target (Mouse) Clone Number Cat# Supplier
113 In Thy1.2 30-H12 105333 Biolegend
115In TER119 TER-119 116241 Biolegend
141Pr Ly6G 1A8 127637 Biolegend
142Nd CD11c N418 3142003B Fluidigm
143Nd TCRb H57-597 3143010B Fluidigm
144Nd CD24 M1/69 101829 Biolegend
146Nd F4/80 BM8 3146008B Fluidigm
148Nd CD11b M1/70 3148003B Fluidigm
149Sm CD19 6D5 3149002B Fluidigm
151Eu CD25 3C7 3151007B Fluidigm
152Sm SiglecF S17007L 155502 Biolegend
160Gd CD62L MEL-14 3160008B Fluidigm
162Dy Ly6C HK1.4 3162014B Fluidigm
168Er CD8a 53-6.7 3168003B Fluidigm
170Er NK1.1 PK136 3170002B Fluidigm
171Yb CD44 IM7 3171003B Fluidigm
172Yb CD4 RM4-5 3172003B Fluidigm
174Yb MHCII M5/114.15.2 3174003B Fluidigm
176Yb B220 RA3-6B2 3176002B Fluidigm

8.Others: Isotype control antibody Cat# MAB002, Clone#11711, R&D Systems Inc., final concentration 0.16 μg/μl
anti-human CCL5 antibody Cat#MAB2781 Clone#16411, R&D Systems Inc., final concentration 0.16 μg/μl

Validation

1. Rat anti mouse CD68 antibody, clone FA-11, from Bio-rad has been used in many mouse models for the identification of CD68 in immunohistochemical studies, using both frozen and paraffin-embedded tissues (Masaki et al. 2003) and (Devey et al. 2009), as stated in the manufacturer's website. The antibody was previously characterized in peer-reviewed publications (e.g. Ramprasad, M.P. et al. 1996, Rabinowitz, S.S. & Gordon, S. 1991, Jayagopal, A. et al. 2009), as stated in the manufacturer's website.
2. Mouse anti-Rabbit Macrophage, RAM11 has been quality validated by Agilent Dako by immunohistochemistry using the LSAB2 system to immunostain formalin-fixed, paraffin-embedded rabbit macrophage-positive tissue sections, as stated in the manufacturer's website. The antibody was previously characterized in peer-reviewed publications (e.g. Tsukada T, et al. 1986, Rosenfeld ME, et al. 1991, O'Brien K, et al. 1991) as referenced in the manufacturer's website.
4. Rabbit anti-mouse CD3, has been quality validated by Thermo Scientific by immunohistochemistry in frozen tissues and paraffin as stated in the manufacturer's website. The antibody was previously characterized in peer-reviewed publications (e.g. Jiang Li, et al. 2012, Kaukinen K., et al. 2007, Zamo' A., et al. 2007)
7. Cytof antibodies dilutions: CyTOF antibodies: a full list is provided in supplementary table 3 for the human CyTOF panel and Supplementary Table 4 for the mouse panel. Antibodies were used at a concentration of 0.2-0.5ug/ ml or 1 test for 1x106 live cells in 100 μl, as per maufacturer's instructions. All antibodies were validated at the Human Immune Monitoring Center of the Icahn School of Medicine at Mount Sinai and at Fluidigm and they were internally controlled by comparison to previous batches of antibodies used at the IMC at Mount Sinai.
8. Isotype control antibody Cat#MAB002 and anti-human CCL5 Cat#MAB2781 were used in many peer reviewed articles (i.e: MAB002, S Qiao et Nat. Comm. 2023 and MAB2781, Z Zheng et al, J. Immunol. 2018).

Animals and other research organisms

Policy information about studies involving animals; ARRIVE guidelines recommended for reporting animal research, and Sex and Gender in Research

Laboratory animals	- 6 weeks old ApoE-/- male mice were used for mouse work and randomized to follow atherosclerosis progression.<br>- 16 weeks old C57BL/6 male mice were used for mouse work for seahorse assays.<br>- 3 months old New Zealand White male rabbits were used for rabbit work and randomized to follow atherosclerosis regression.<br>Housing conditions: 12-hour light/12-hour dark cycle conditions; temperature 20-24°C, 30-70% humidity.
Wild animals	No wild animals were used in this study.
Reporting on sex	Human studies, patients: 52.9% males, females 47.1%; Healthy donors: males 46% and females 54%.<br>Mouse: only males<br>Rabbits: only males
Field-collected samples	No field-collected samples were used in this study.
Ethics oversight	The mouse and the rabbit work was performed under the study protocol IACUC-2016-0032, as approved by the Institutional Animal Care and Use Committee of the Icahn School of Medicine at Mount Sinai. Additional mouse work was performed under the study protocol PROTO202100030, as approved by the Institutional Animal Care and Use Committee of the NYU Grossman School of Medicine.

Note that full information on the approval of the study protocol must also be provided in the manuscript.
